# ΔNp63α promotes Epstein-Barr virus latency in undifferentiated epithelial cells

**DOI:** 10.1371/journal.ppat.1010045

**Published:** 2021-11-08

**Authors:** Nicholas Van Sciver, Makoto Ohashi, Dhananjay M. Nawandar, Nicholas P. Pauly, Denis Lee, Kathleen R. Makielski, Jillian A. Bristol, Sai Wah Tsao, Paul F. Lambert, Eric C. Johannsen, Shannon C. Kenney

**Affiliations:** 1 Department of Oncology, School of Medicine and Public Health, University of Wisconsin- Madison, Madison, Wisconsin, United States of America; 2 Currently at Ring Therapeutics, Cambridge, Massachusetts, United States of America; 3 School of Biomedical Sciences, Li Ka Shing Faculty of Medicine, The University of Hong Kong, Hong Kong, China; 4 Department of Medicine, School of Medicine and Public Health, University of Wisconsin-Madison, Madison, Wisconsin, United States of America; University of Utah, UNITED STATES

## Abstract

Epstein-Barr virus (EBV) is a human herpesvirus that causes infectious mononucleosis and contributes to both B-cell and epithelial-cell malignancies. EBV-infected epithelial cell tumors, including nasopharyngeal carcinoma (NPC), are largely composed of latently infected cells, but the mechanism(s) maintaining viral latency are poorly understood. Expression of the EBV BZLF1 (Z) and BRLF1 (R) encoded immediate-early (IE) proteins induces lytic infection, and these IE proteins activate each other’s promoters. ΔNp63α (a p53 family member) is required for proliferation and survival of basal epithelial cells and is over-expressed in NPC tumors. Here we show that ΔNp63α promotes EBV latency by inhibiting activation of the BZLF1 IE promoter (Zp). Furthermore, we find that another p63 gene splice variant, TAp63α, which is expressed in some Burkitt and diffuse large B cell lymphomas, also represses EBV lytic reactivation. We demonstrate that ΔNp63α inhibits the Z promoter indirectly by preventing the ability of other transcription factors, including the viral IE R protein and the cellular KLF4 protein, to activate Zp. Mechanistically, we show that ΔNp63α promotes viral latency in undifferentiated epithelial cells both by enhancing expression of a known Zp repressor protein, c-myc, and by decreasing cellular p38 kinase activity. Furthermore, we find that the ability of cis-platinum chemotherapy to degrade ΔNp63α contributes to the lytic-inducing effect of this agent in EBV-infected epithelial cells. Together these findings demonstrate that the loss of ΔNp63α expression, in conjunction with enhanced expression of differentiation-dependent transcription factors such as BLIMP1 and KLF4, induces lytic EBV reactivation during normal epithelial cell differentiation. Conversely, expression of ΔNp63α in undifferentiated nasopharyngeal carcinoma cells and TAp63α in Burkitt lymphoma promotes EBV latency in these malignancies.

## Author summary

Epstein-Barr virus (EBV) is an important cause of both epithelial cell and B cell human cancers. EBV-infected tumors have predominantly latent viral infection, allowing them to escape the cell killing that occurs during lytic viral infection. EBV is highly lytic in normal differentiated oral epithelial cells. Thus, an important question is how the virus maintains the latent form of viral infection in EBV-associated epithelial cell tumors such as undifferentiated nasopharyngeal carcinoma (NPC). This study demonstrates that the cellular transcription factor ΔNp63ɑ, which is specifically expressed in undifferentiated basal epithelial cells and is over-expressed in NPC tumors, maintains EBV latency by inhibiting the activity of the viral immediate-early (IE) promoter (Zp) that drives expression of the BZLF1 IE protein. A related splice variant, TAp63α, found in some EBV+ lymphomas, has a similar inhibitory effect. Our findings reveal that ΔNp63ɑ and TAp63α contribute to EBV latency in both epithelial and B cell tumors. Furthermore, since differentiation results in loss of ΔNp63ɑ expression, our results help to explain why lytic EBV reactivation is promoted by epithelial cell differentiation.

## Introduction

Epstein-Barr virus (EBV) is a gamma-herpesvirus that infects over 90% of the human population by adulthood and causes the clinical syndrome, infectious mononucleosis. EBV primarily infects B cells and epithelial cells and is associated with both B-cell and epithelial-cell malignancies, including Burkitt lymphoma (BL), diffuse large B cell lymphoma, Hodgkin lymphoma, gastric carcinoma, and nasopharyngeal carcinoma (NPC) [1]. Like all herpesviruses, EBV can infect host cells in either latent or lytic forms and persists in the host for life. In latently infected cells, the virus expresses only a small subset of viral genes and replicates once per cell cycle using the host cell DNA polymerase. In lytically infected cells, the full viral gene complement is expressed, the virus is replicated using the virally-encoded DNA polymerase and infectious virions are produced. The major site of persistent latent EBV infection in humans is memory B cells, although the lytic form of infection can be induced by B-cell receptor stimulation, plasma cell differentiation or various other stimuli in B cells [2–6].

In contrast, normal oropharyngeal epithelial cells support the lytic form of EBV infection, and it remains uncertain whether latent EBV infection of epithelial cells normally occurs in humans [7–9]. Lytically-infected epithelial cells are thought to be the major source of infectious EBV particles in saliva [10] and thus are essential for the spread of the virus from host to host. EBV infection of non-transformed tongue epithelial cells causes the clinical syndrome, oral hairy leukoplakia (OHL), in immunocompromised patients [7,11]. Analysis of OHL lesions has revealed that EBV infection is confined to the more differentiated epithelial cell layers, and that the infection is completely lytic without evidence of concomitant latent infection [7,11]. Nevertheless, the EBV-associated epithelial tumor, undifferentiated nasopharyngeal carcinoma, contains largely latent EBV infection, and the ability of the virus to maintain latency in the tumor cells is likely required for NPC development. Thus, understanding how EBV stays latent in a cell type where it is usually lytic is important for understanding NPC.

Lytic EBV infection is initiated by expression of the two EBV immediate-early (IE) proteins, BZLF1 (Z) and BRLF1 (R) [12–14]. The Z and R IE genes are driven by the Z and R promoters, respectively, and activation (or repression) of these promoters by cellular transcription factors serves as the major control point in determining if EBV infection is latent versus lytic in cells [15–22]. Once expressed, the Z and R proteins function as viral transcription factors that activate each other’s promoters, as well as their own promoters, in a positive feedback loop [12,23–25]. Z and R then synergistically activate early lytic viral gene promoters to induce early gene expression and lytic viral DNA replication.

Z is a part of the bZip protein family and binds to AP-1-like sites called Z-responsive elements (ZREs), while R has no cellular homolog and binds to R-responsive elements (RREs) [12,26,27]. R can also activate some promoters, including Zp, indirectly through non-DNA binding mechanisms that are still not totally understood [25,28]. Many ZREs are preferentially bound by Z in the methylated form, helping Z to induce lytic viral reactivation even when the viral genome becomes highly methylated (as occurs normally in EBV-infected B cells and in EBV-positive B cell and epithelial cell tumors)[29]. In contrast, R preferentially activates unmethylated lytic EBV promoters [29], and in EBV-infected hTERT-immortalized normal oral keratinocyte cells (NOKs), where the viral genome remains hypomethylated, over-expression of R, but not Z, induces lytic reactivation [12,13,29–32]. Regardless of the viral genome methylation state, Z and R synergistically activate many early lytic gene promoters and expression of both proteins is required for the virus to lytically replicate and complete the lytic cycle [29,33].

We and others have previously demonstrated that epithelial cell differentiation induces EBV lytic reactivation in stably infected oral keratinocyte cells [15,16,34–39]. Similarly, EBV infection of primary oral epithelial cells grown in stratified “raft” cultures resulted in no detectable latent or lytic infection in undifferentiated layers and a robust lytic infection in the differentiated cell layers [8]. We show the cellular transcription factors KLF4 and BLIMP1, which are preferentially expressed in differentiated epithelial cells, synergistically activate the Z, R, and LMP1 EBV promoters, at least partly explaining why EBV reactivation is induced by epithelial cell differentiation [15,16,36,40,41]. However, given the robust latency of EBV infection in undifferentiated epithelial cell models [16,34–36], we hypothesized that additional factors expressed in undifferentiated (but not differentiated) epithelial cells may also contribute to the differentiation-dependent phenotype by inhibiting lytic viral reactivation in undifferentiated cells.

Here we show that expression of the master-regulator of epithelial cell identity, p63, in undifferentiated epithelial cells is a potent negative regulator of lytic EBV reactivation. The cellular p63 protein is a member of the p53 family that transcriptionally regulates many different epithelial cell-specific genes [42]. The major epithelial cell p63 isoform, ΔNp63ɑ, is a critical regulator of proliferation and differentiation in keratinocytes and a marker for the epithelial stem cell compartment [42–47]. ΔNp63ɑ mutations result in developmental defects in limb development, while deletion of ΔNp63ɑ in mice is lethal after birth due to a lack of a stratified epidermis [42,46,48]. Like p53, p63 contains a DNA binding domain and, through this domain, can bind to thousands of sites on the human genome to regulate transcriptional function [49,50]. This DNA binding domain retains homology to p53, and p63 can bind to some of the same promoter sites as p53 [51]. However, unlike p53, ΔNp63ɑ is rarely mutated in cancer and instead has been reported to be over-expressed in several epithelial tumor types, including NPC [52,53]. We find that ΔNp63ɑ inhibits lytic EBV reactivation in epithelial cells by decreasing activity of the Z IE promoter and show that this effect is mediated both by reduced cellular p38 kinase activity and increased c-myc expression. In addition, we show that another isoform of p63, TAp63ɑ, which is expressed in some EBV+ B cell lymphomas, likewise inhibits EBV reactivation. These findings not only further elucidate how EBV uses differentiation-dependent transcription factors (ΔNp63ɑ, KLF4 and BLIMP1) to ensure differentiation-dependent lytic reactivation in epithelial cells, but also reveal an important mechanism for promoting viral latency in EBV-infected tumor cells.

## Results

### ΔNp63α and immediate-early protein Z are expressed in distinct sections of organotypic raft cultures of NOKs-Akata cells

**Fig 1:**
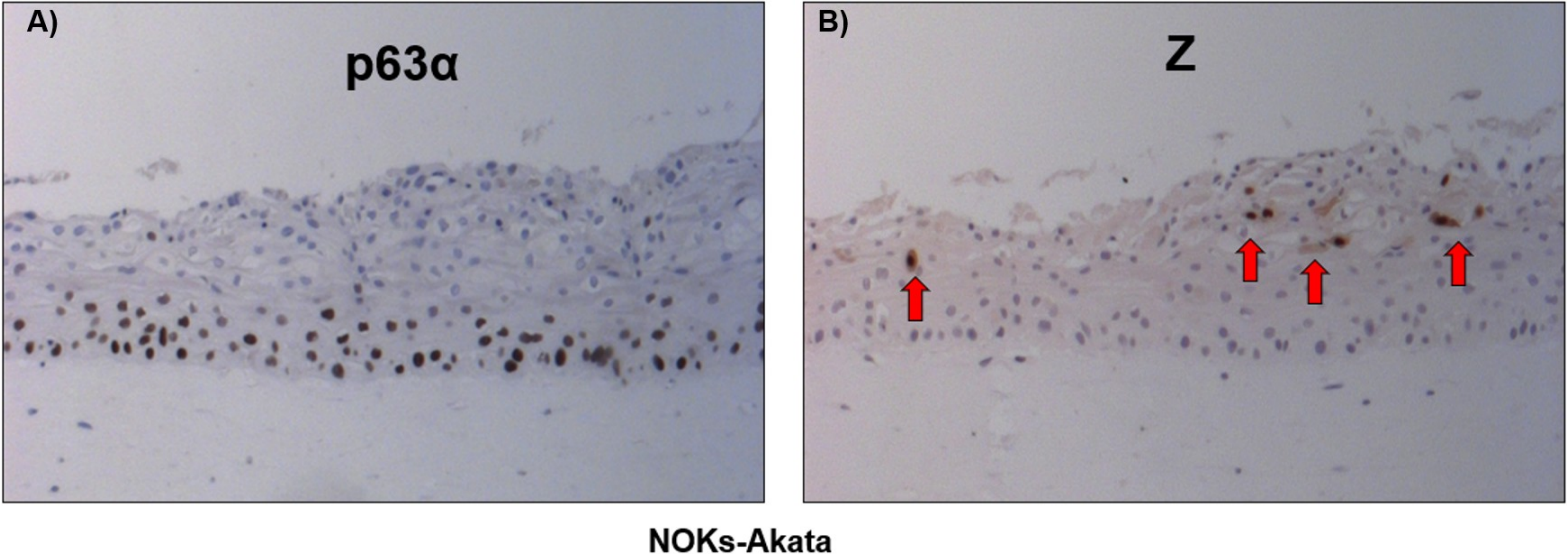
ΔNp63α and Z expression occurs in different sections of organotypic raft cultures. Immunohistochemistry (IHC) was performed on adjacent sections of organotypic rafts of differentiated NOKs-Akata cells using antibodies against **A)** p63ɑ and **B)** Z. Arrows show examples of Z expressing cells. Results are representative of three independent experiments.

Since ΔNp63ɑ is the master regulator of stem cell identity in undifferentiated basal epithelial cells, and inhibits epithelial cell differentiation [43,54], we hypothesized that it also blocks lytic EBV reactivation. NOKs-Akata is an EBV-infected telomerase-immortalized normal oral keratinocyte cell line that retains the ability to differentiate. We previously showed that lytic reactivation of EBV occurs only in the differentiated layers of rafted NOKs-Akata cells [16,34,36]. In contrast, ΔNp63ɑ is primarily expressed in the basal epithelial layer of normal stratified epithelium. ΔNp63ɑ expression is lost during epithelial cell differentiation due to IRF6-mediated protein degradation as well as the antagonistic effect of the differentiation-dependent miR-203 [55–58]. To determine if expression of the IE lytic EBV protein Z overlaps that of ΔNp63ɑ in rafted NOKs-Akata cells, we performed immunohistochemistry (IHC) using anti-Z or anti-p63ɑ antibodies. ΔNp63ɑ was expressed primarily in the basal layer and immediate suprabasal layers of the NOKs-Akata cell raft cultures, and ΔNp63ɑ expression ceased in the more differentiated layers, congruent with previous literature [55–57] (**Fig 1A**). In contrast, Z expression was observed exclusively in the raft’s more differentiated layers (**Fig 1B**) with no overlap in expression of the ΔNp63ɑ and Z proteins in the raft cultures. These observations are consistent with the hypothesis that ΔNp63ɑ impairs the ability of EBV to lytically reactivate in epithelial cells.

### Knockdown of ΔNp63α expression results in EBV lytic reactivation in EBV+ carcinoma and immortalized keratinocyte cell lines

**Fig 2:**
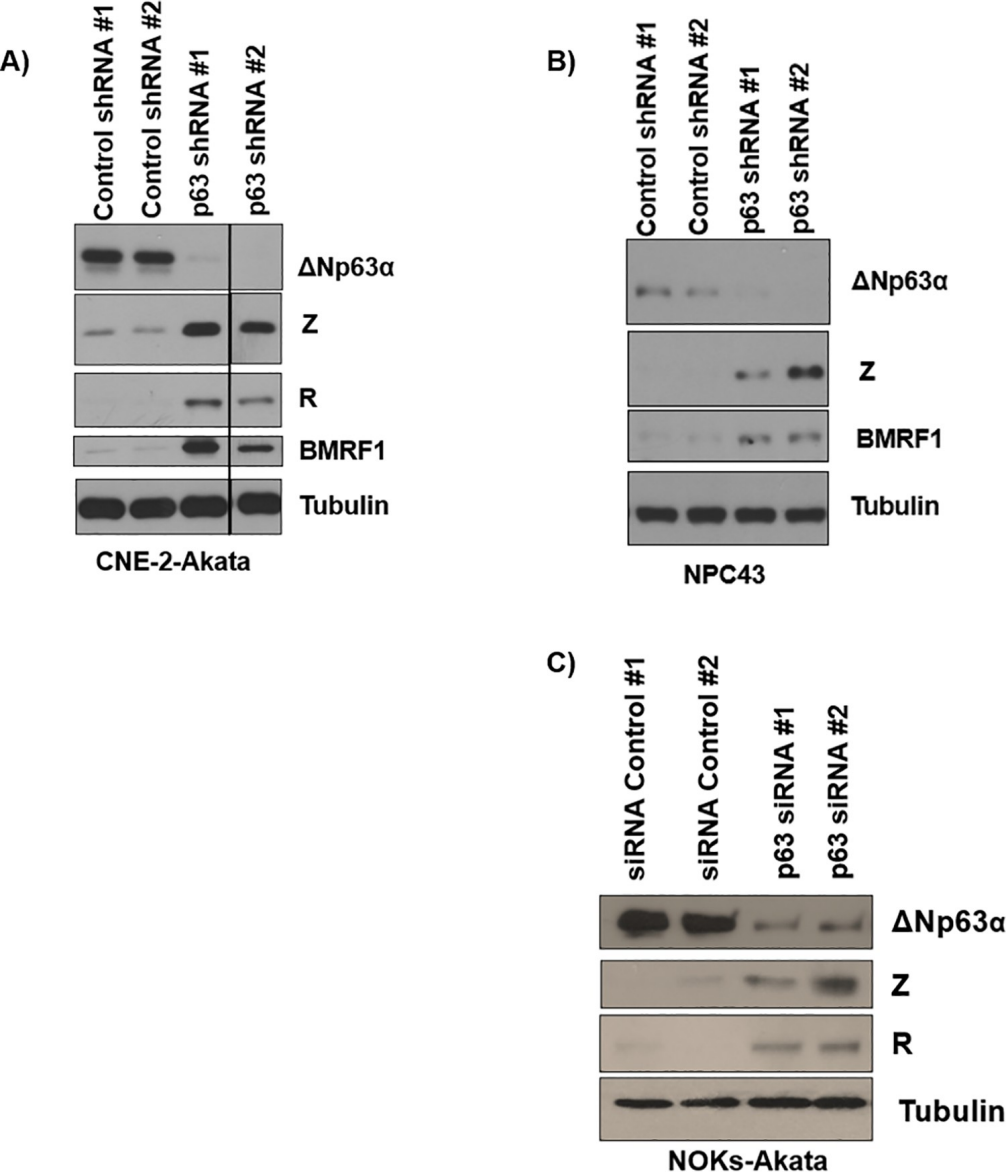
ΔNp63α depletion results in EBV lytic reactivation in EBV+ carcinomas (CNE-2 and NPC43) and telomerase-immortalized oral keratinocyte (NOKs) cell lines. **A)** CNE-2-Akata cells were infected with lentiviruses expressing shRNAs against p63 or a control sequence, selected with puromycin, and harvested for immunoblot analyses to measure expression levels of the EBV Z, R, and BMRF1 lytic proteins, ΔNp63ɑ, and tubulin proteins as indicated. **B**) NPC43 cells were infected with lentiviruses expressing shRNAs that target p63, or a control sequence. Two days post-infection, cells were harvested for immunoblot analysis to assess the expression of Z, BMRF1, ΔNp63ɑ, and the loading control tubulin. **C)** NOKs-Akata cells were transfected with siRNAs against p63 or a control siRNA. Two days after transfection, the cells were harvested for immunoblot analyses and expression of the Z, R, ΔNp63ɑ and tubulin proteins was examined as indicated. The black line indicates where irrelevant lane(s) were removed. The original western blots used to construct this figure are shown in supplemental Fig 1. All figures shown are representative of eight independent biological replicates.

To determine if ΔNp63ɑ expression inhibits EBV lytic reactivation in the context of undifferentiated epithelial cells, lentivirus vectors expressing shRNAs targeting p63 (or control vectors) were used to infect the CNE-2-Akata cell line (a carcinoma cell line incapable of differentiation) and stably infected cells were selected with puromycin. As shown in **Fig 2A**, ΔNp63ɑ expression was successfully decreased with the p63 targeting shRNAs. Furthermore, loss of ΔNp63ɑ expression resulted in increased expression of EBV IE proteins Z and R as well as the early lytic protein BMRF1 in comparison to control lentivirus infections (**Fig 2A**). Importantly, we confirmed that depletion of ΔNp63ɑ in an authentic EBV-infected nasopharyngeal cell line, NPC43 [59], also induced expression of the lytic viral proteins Z and BMRF1 (**Fig 2B**).

Additionally, siRNAs targeting ΔNp63ɑ were delivered into a third EBV-infected epithelial cell line, NOKs-Akata cells, which retain the ability to differentiate [16]. As shown in **Fig 2C**, knockdown of ΔNp63ɑ expression also increased expression of EBV IE proteins, Z and R, in NOKs-Akata cells. These results indicate ΔNp63ɑ expression represses the EBV lytic cascade and that this repression can occur even in carcinoma cell lines that are incapable of differentiation.

### ΔNp63ɑ inhibits lytic reactivation during epithelial cell differentiation

**Fig 3:**
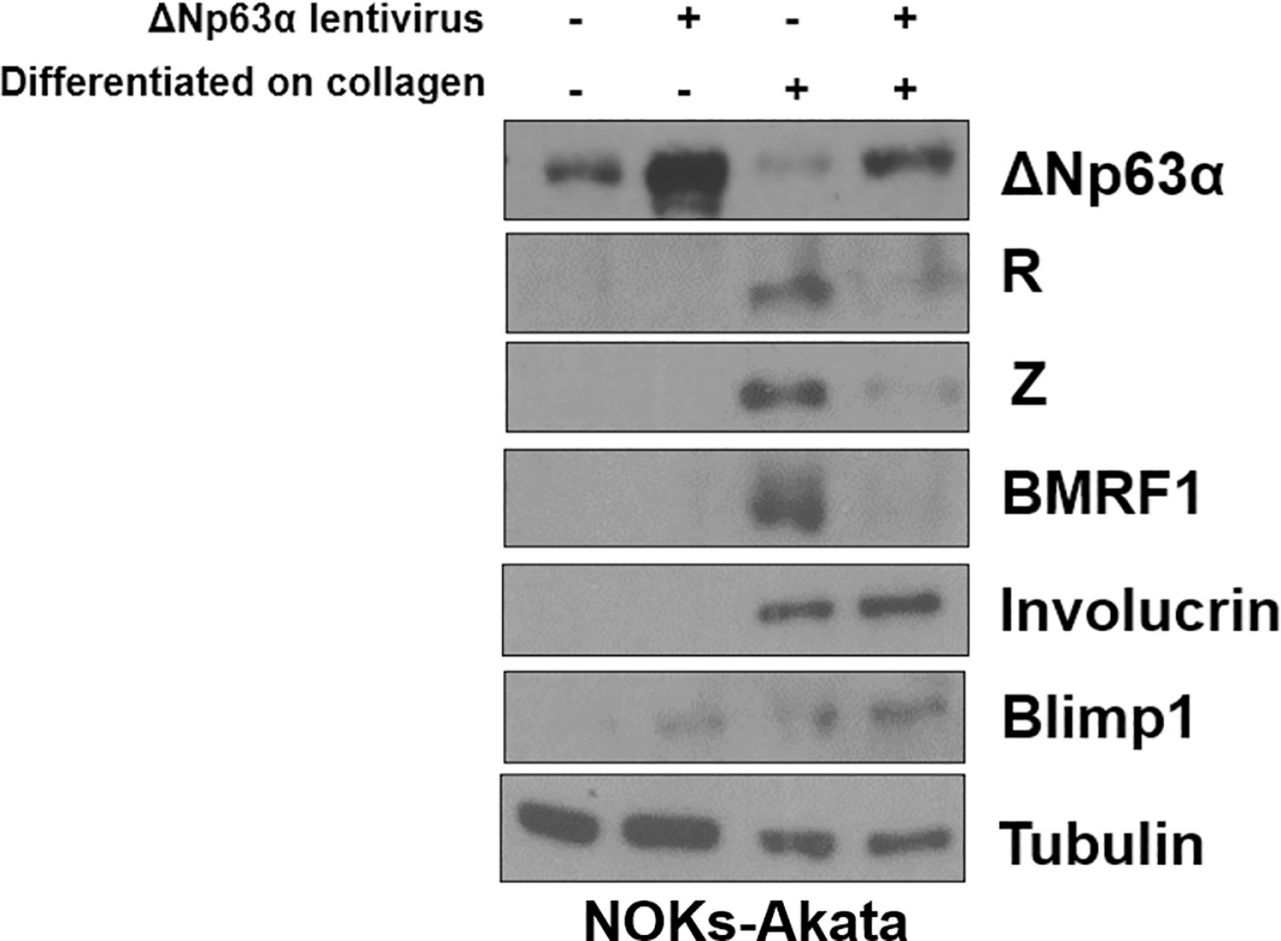
ΔNp63α over-expression in NOKs-Akata cells during differentiation decreases EBV lytic reactivation. NOKs-Akata cells expressing either ΔNp63ɑ from a lentivirus or infected with a control lentivirus were differentiated on collagen-treated membranes in air-liquid interface culture for three days in the presence of media containing vitamin C, calcium, and 10% serum. The cells were then harvested for an immunoblot and expression levels of the R, Z, BMRF1, ΔNp63ɑ, involucrin, BLIMP1, and tubulin proteins was determined as indicated.

EBV lytically reactivates during epithelial cell differentiation, and this effect is at least partially mediated through enhanced expression of the transcription factors KLF4 and BLIMP1 [15,16,36]. ΔNp63ɑ is known to impede keratinocyte differentiation through transcriptional repression of cellular genes required for differentiation [54,60,61]. To determine if ΔNp63ɑ over-expression reduces EBV lytic reactivation during epithelial cell differentiation, we stably infected NOKs-Akata cells with either a lentivirus over-expressing ΔNp63ɑ or with a control lentivirus vector, and then seeded the cells onto collagen-treated membranes in air-liquid interface culture conditions. After three days of differentiating the cells, immunoblot analysis was performed to assess EBV lytic reactivation and cellular differentiation. While EBV lytically reactivated in the NOKs-Akata cells infected with the control lentivirus, cells infected with the ΔNp63ɑ expressing lentivirus had reduced expression of the lytic EBV proteins, R, Z, and BMRF1 (**Figs 3 and S3**). Interestingly, the levels of the differentiation-induced cellular proteins, involucrin and BLIMP1, did not change between cells infected with the control and ΔNp63ɑ- expressing lentiviruses, indicating that ΔNp63ɑ repression of EBV lytic reactivation may be directly impeding viral processes as opposed to reducing cellular differentiation in these experiments.

### ΔNp63α over-expression inhibits R- but not Z- mediated induction of lytic reactivation

**Fig 4:**
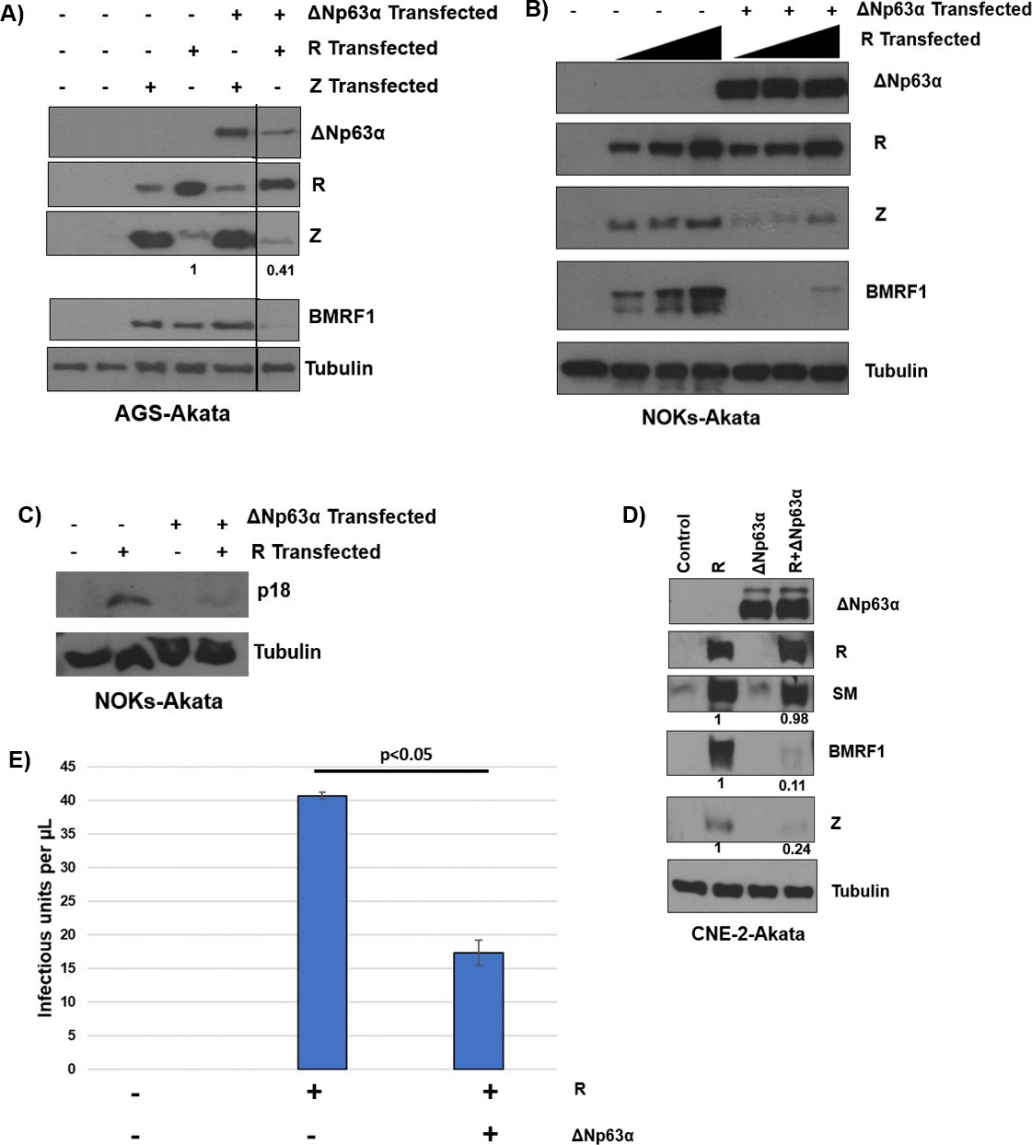
ΔNp63α over-expression inhibits R-mediated, but not Z-mediated, lytic reactivation. **A)** AGS-Akata cells were transfected with Z or R expression vectors, with or without a co-transfected ΔNp63ɑ expression vector. Immunoblots were performed to assess expression levels of the EBV lytic proteins Z, R, and BMRF1 as indicated, as well as the loading control, tubulin. Z expression was quantitated by ImageJ software, normalized to tubulin and the Z alone condition was set as 1. **B)** NOKs-Akata cells were transfected with 5 ng, 10 ng, or 50 ng of an R expression vector with or without a co-transfected ΔNp63ɑ expression vector. Immunoblot was performed to examine expression levels of the EBV lytic proteins Z, R, and BMRF1, as well as ΔNp63ɑ and tubulin as indicated. Note that the western shown is a very short exposure to only capture expression of transfected (but not endogenous) ΔNp63ɑ. **C)** NOKs-Akata cells were transfected with 10 ng of an R expression vector with or without a co-transfected ΔNp63ɑ expression vector or the ΔNp63ɑ expression vector alone (same extracts as in Fig 4B) and an immunoblot was performed to examine expression of the late viral protein, viral capsid protein p18. Tubulin was used as a loading control. **D)** CNE-2-Akata cells were transfected with an R expression vector in the presence or absence of a co-transfected ΔNp63ɑ vector. An immunoblot was performed to examine expression levels of the ΔNp63ɑ, R, Z, BMRF1, SM, and tubulin proteins as indicated. SM, BMRF1, and Z expression was quantified using ImageJ, normalized to tubulin, with the R transfected by itself set as 1. The black line in Fig 4A shows where irrelevant lane(s) were removed; the original western blots used to construct Fig 4A are shown in supplemental Fig 4. The short exposure shown detects expression of transfected (but not endogenous) ΔNp63ɑ. **E)** CNE-2-Akata cells were transfected with control vector, an R expression vector, or R plus ΔNp63ɑ expression vectors. Three days after transfection infectious virus titers in each condition were quantitated as described in the methods. All results depicted have been independently confirmed a minimum of two times.

To begin to examine the mechanism(s) for the ΔNp63ɑ effect on lytic EBV reactivation, we next compared the effect of co-transfected ΔNp63ɑ protein on the ability of the Z versus R EBV IE proteins to induce lytic EBV reactivation when transfected into EBV-infected epithelial cell lines that can be reactivated by either Z or R transfection. In the gastric carcinoma cell line AGS-Akata, which lacks endogenous ΔNp63ɑ expression, co-transfected ΔNp63ɑ inhibited the ability of transfected R protein to induce expression of endogenously expressed lytic viral proteins, Z and BMRF1 (**Fig 4A)**. In contrast, co-transfected ΔNp63ɑ protein did not inhibit the ability of transfected Z protein to induce expression of endogenous lytic proteins R and BMRF1 (**Fig 4A**) even though more ΔNp63ɑ was present in this condition. A repeat experiment confirmed that ΔNp63ɑ does not inhibit the ability of transfected Z to induce lytic protein expression in AGS-Akata cells (**S5 Fig**). This result suggests that ΔNp63ɑ may inhibit lytic EBV reactivation through effects on the R IE protein function and/or the BZLF1 Z promoter, since R must first induce Z expression in order to turn on early lytic genes such as BMRF1 [33].

To confirm that ΔNp63ɑ inhibits R-mediated lytic reactivation, we examined its effect in NOKs-Akata cells, in which R transfection, but not Z transfection, can initiate the lytic cascade [29]. Similar to the results in AGS-Akata cells, ΔNp63ɑ co-transfection inhibited the ability of transfected R protein to induce expression of the Z and BMRF1 proteins, as well as the late viral capsid protein, p18 (**Fig 4B and 4C**). Co-transfected ΔNp63ɑ likewise decreased the ability of transfected R to induce expression of the Z and BMRF1 lytic proteins in CNE-2-Akata cells (**Fig 4D**).

While R activates many lytic EBV promoter targets through a direct DNA binding mechanism [62], it is not known to bind directly to the IE Z promoter and instead is thought to activate this promoter through indirect effects on cellular transcription factors [24,25,63,64]. In addition, while some early lytic EBV promoters (such as the BMRF1 promoter) contain both Z and R binding sites and require both Z and R for efficient expression [29,65,66], maximal expression of the SM early lytic promoter has been shown to only require R binding to the promoter, and is not further enhanced by Z expression [67,68]. Interestingly, we found that while ΔNp63ɑ inhibits the ability of co-transfected R to activate expression of the Z and BMRF1 proteins in CNE-2-Akata cells, it does not affect R activation of the SM protein (**Fig 4D**) [62,67]. Finally, we found that co-transfection of R with ΔNp63ɑ in CNE-2-Akata cells decreased the production of infectious virions compared to R transfected alone (**Fig 4E**). These results suggest that ΔNp63ɑ primarily inhibits lytic EBV reactivation by preventing R’s ability to induce the Z IE promoter, since the concomitant decreased expression of the BMRF1 and VCA p18 proteins, as well as the decreased titer of infectious virions, would both be the expected outcome from loss of Z expression.

### ΔNp63α repression of EBV lytic reactivation is independent of p53

**Fig 5:**
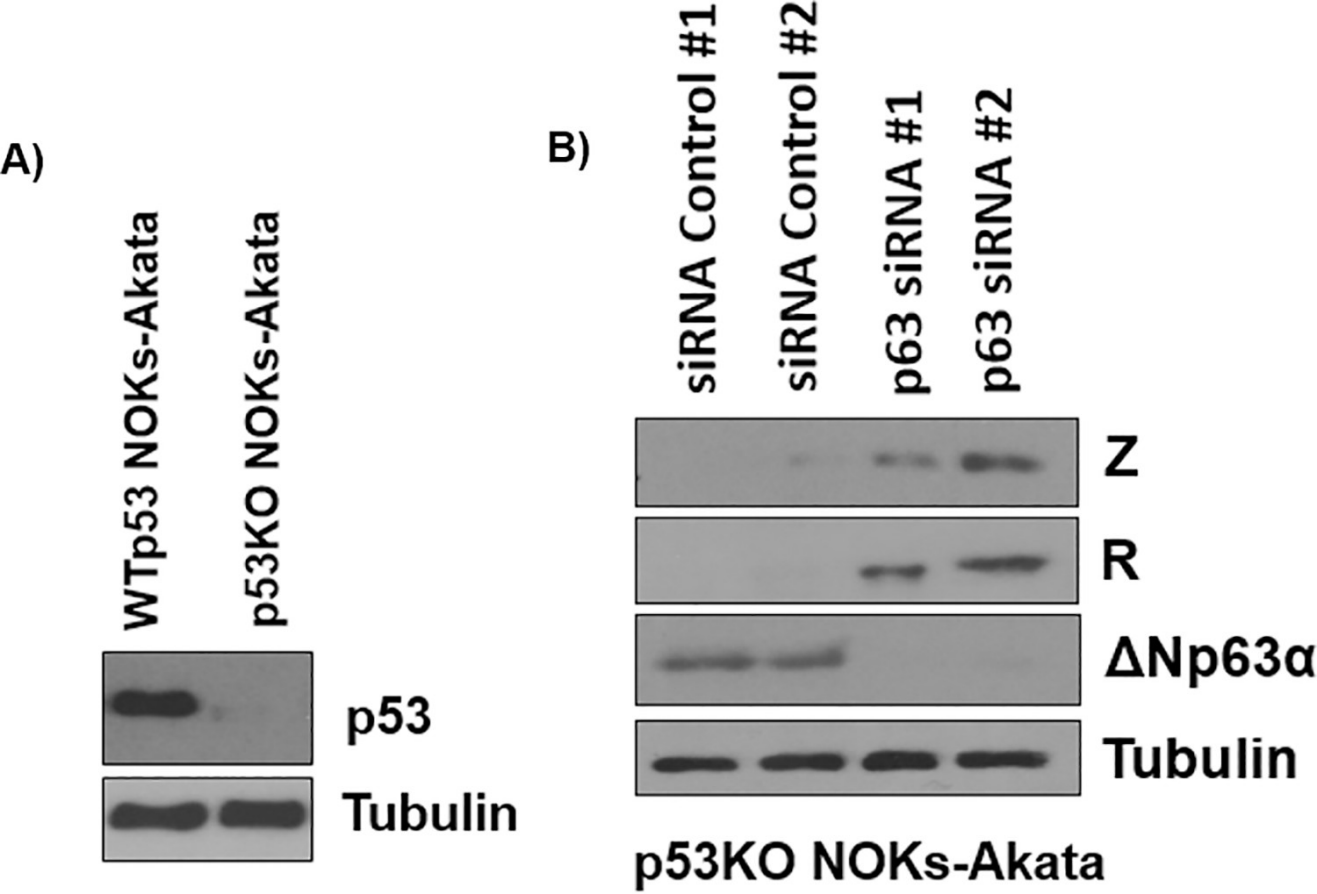
ΔNp63 inhibits lytic reactivation in NOKs-Akata cells even when the p53 gene is deleted. **A)** The CRISPR/CAS9 technique was used to knock out p53 expression in NOKs-Akata cells, as described previously [73], and expression of the p53 protein and tubulin loading control was examined by immunoblot to confirm knock-out of p53 expression. **B)** NOKs-Akata cells in which the p53 gene was knocked-out were transfected with siRNAs against ΔNp63ɑ or a control sequence. Two days later expression of the Z, R, ΔNp63ɑ and tubulin proteins was examined by immunoblot. These results are representative of three biological replicate experiments.

ΔNp63ɑ is a member of the p53 protein family, and approximately 60% of ΔNp63ɑ’s DNA binding domain is homologous to the DNA binding domain of p53 [55]. Furthermore, p53 and ΔNp63ɑ bind to some of the same promoters, and ΔNp63ɑ can repress p53 activation at these promoters [51,55,69]. Since p53 was previously reported to be a positive regulator of EBV lytic reactivation and binds to the Z promoter via the Sp1 transcription factor and HIF-1α [70–73], we determined if ΔNp63ɑ’s repression of EBV lytic reactivation is due to competition with p53. For these experiments, ΔNp63ɑ expression was inhibited by siRNAs in a NOKs-Akata cell line that had CRISPR-Cas9 mediated knockout of p53 (**Figs 5A and S6**). As shown in **Fig 5B**, immunoblot analysis revealed that loss of ΔNp63ɑ expression upregulates Z and R expression even in the absence of p53 expression. These results suggest that ΔNp63ɑ represses EBV lytic reactivation independently of competitive inhibition of p53 in EBV infected cells.

### ΔNp63ɑ does not directly bind to the EBV immediate-early promoters

**Fig 6:**
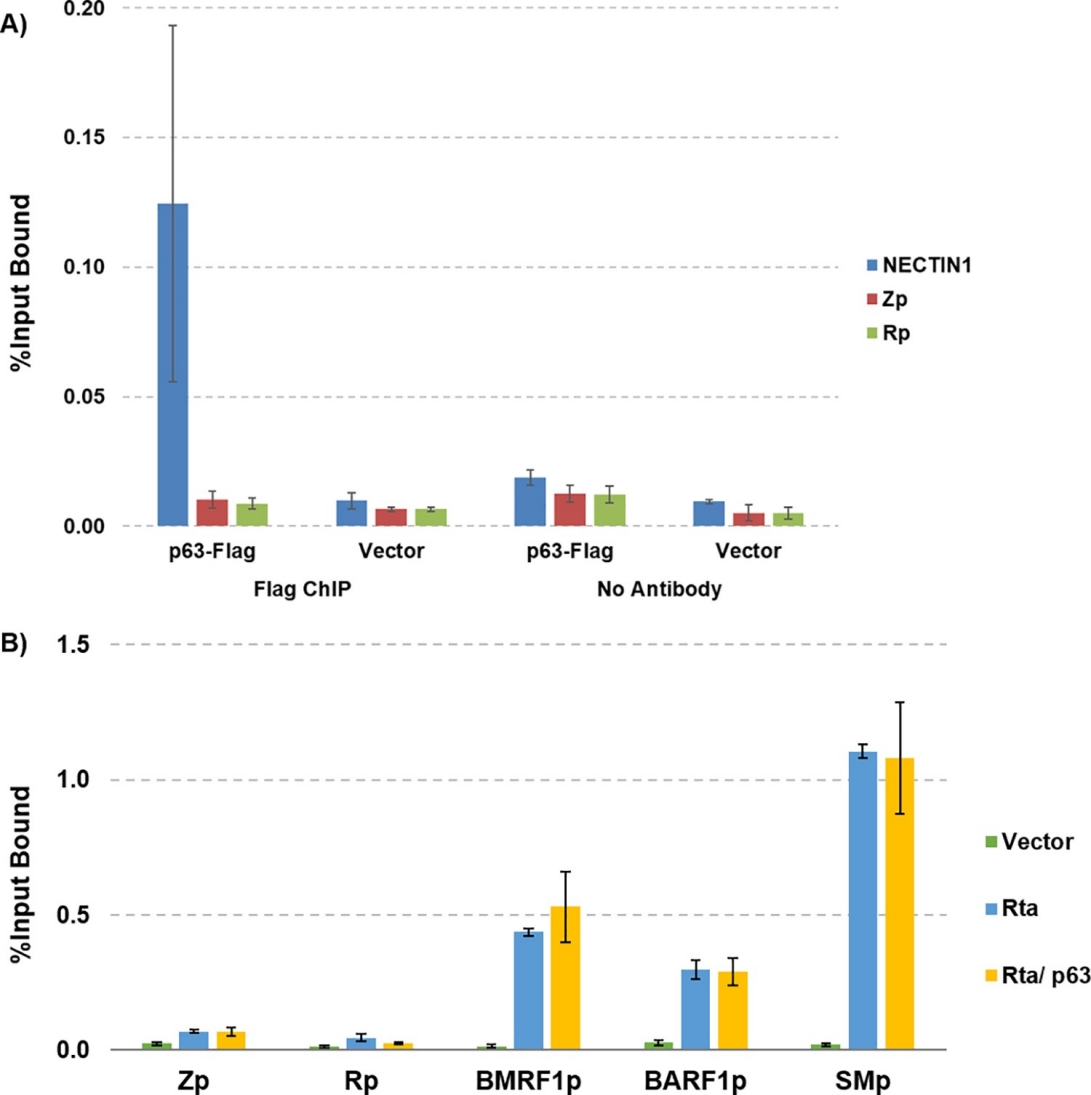
ΔNp63α does not bind directly to the EBV immediate-early gene promoters or prevent R binding to the EBV genome. **A)** AGS-Akata cells were transfected with a FLAG-tagged ΔNp63ɑ vector or a control vector, and two days after transfection a ChIP assay was performed as described in the methods using an anti-FLAG antibody. qPCR was conducted to determine ΔNp63ɑ occupancy of EBV lytic gene IE promoters Zp and Rp, in addition to NECTIN1, a known ΔNp63ɑ binding site. **B)** CNE-2-Akata cells were transfected with vector control or with a FLAG-tagged R expression vector in the presence or absence of ΔNp63ɑ, and a ChIP assay was performed as described in the methods using an anti-FLAG antibody. qPCR was performed in the ChIP samples to examine R association with the EBV Z promoter, R promoter, BMRF1 promoter, BARF1 promoter, and BMLF1 (SM) promoter. The experiments shown are representative of two independent experiments and the error bar indicates standard error of the mean within experiments.

ΔNp63ɑ can function as either a transcriptional repressor or transcriptional activator, and is known to bind to promoters of cellular genes such as KLF4, p21, and p16 to prevent their expression and maintain proliferation of the keratinocyte stem cell compartment [61,69,74]. To determine if ΔNp63ɑ inhibits lytic EBV replication by binding directly to the Zp and/or Rp IE viral promoters, we conducted chromatin-immunoprecipitation quantitative PCR (ChIP-qPCR) assays. AGS-Akata cells were transfected with a FLAG-tagged ΔNp63ɑ vector or vector control and ChIP-qPCR assays were performed two days later using an anti-FLAG antibody as described in the methods. As shown in **Fig 6A,** we found that ΔNp63ɑ binds to the cellular promoter for NECTIN1, a known ΔNp63ɑ binding target [75], but we did not detect ΔNp63ɑ binding to either the Z promoter or the R promoter on the EBV genome (**Fig 6A**). This result suggests that ΔNp63ɑ does not bind to the immediate-early gene promoters and inhibits EBV reactivation through a different mechanism.

### ΔNp63α does not prevent R binding to the EBV genome

R binds to RREs on the EBV genome during lytic reactivation to induce lytic gene expression [62]. To determine if ΔNp63α prevents R binding to RREs on the EBV genome during lytic reactivation, CNE-2-Akata cells were transfected with a FLAG-tagged R vector with or without ΔNp63α. We then performed ChIP-qPCR on known RRE sites using primers against the SM (BMLF1) promoter, BMRF1 promoter, BARF1 promoter, and the Z and R promoters (which do not contain RRE sites) to determine if R binding is affected by co-transfection with ΔNp63α [62]. Interestingly, we found that R binds to the EBV genome RRE sites in the SM, BMRF1 and BARF1 promoters equivalently with or without ΔNp63α (**Fig 6B**). These results are consistent with our finding that R activates SM gene expression in the presence or absence of co-transfected ΔNp63α (**Fig 4D**), and again suggest that a major ΔNp63α inhibitory effect may be mediated by decreasing the ability of R to indirectly activate Zp.

### The C-terminal domain of TAp63 and ΔNp63 inhibits R-induced lytic reactivation

There are two primary p63 isoforms, ΔN and TA, which are defined by their N-terminal regions. The isoforms are derived from alternative transcription start sites, which either lack or contain the N-terminal transactivation domain, respectively [42]. An additional five isoform subtypes (α, β, δ, ε, and γ) can also arise from alternative splicing (or in the case of ε, a premature stop codon in codon 10) [55,76,77]. The ΔNp63ɑ isoform is the primary isoform expressed in epithelial cells. Interestingly, TAp63ɑ is expressed in some B-cell lymphomas, including Burkitt lymphoma and diffuse large B cell lymphomas (DLBCL), and is a potential marker for poor patient prognosis [42,55,78–81].

**Fig 7:**
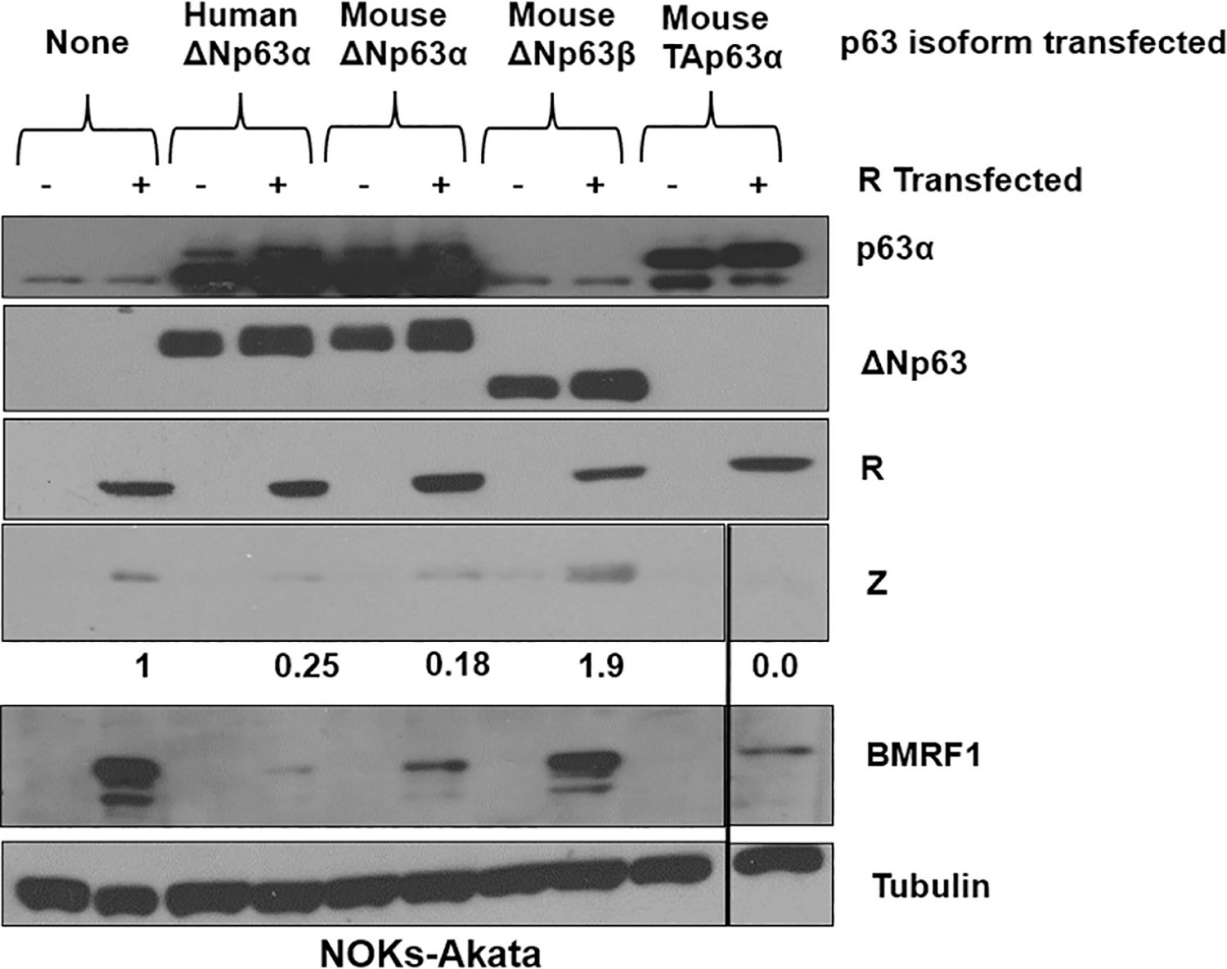
The C-terminus of p63 protein is required for the ability to inhibit R-mediated lytic reactivation. NOKs-Akata cells were transfected with or without an R expression vector in the presence or absence of various different co-transfected p63 isoform vectors, including the human ΔNp63ɑ protein, the mouse ΔNp63ɑ protein, the mouse ΔNp63β isoform (homologous to ΔNp63ɑ but lacking 121 amino acids at the C-terminal domain), or the mouse TAp63α isoform (which is identical to ΔNp63ɑ except for the addition of an N terminal transactivating domain). Two days after transfection, immunoblots were performed to assess expression levels of the Z, R, BMRF1, p63 protein isoforms, and tubulin as indicated. The quantification of Z protein expression was determined through ImageJ, normalized to tubulin, with the R alone condition set as 1. The black lines show where irrelevant lanes were removed; the original western blots used to construct this figure are shown in supplemental Fig 7. This experiment is representative of two independent biological replicates.

To determine the region(s) of ΔNp63 responsible for the suppression of EBV lytic reactivation, we transfected human ΔNp63ɑ, mouse ΔNp63α, and mouse ΔNp63β isoforms into NOKs-Akata cells with or without R. We found that, like human ΔNp63ɑ, mouse ΔNp63α inhibits R-mediated initiation of the lytic replication cycle. However, the mouse ΔNp63β isoform, which is missing 121 amino acids of the C-terminal domain of ΔNp63ɑ, failed to do so (**Fig 7**). Additionally, TAp63α also inhibited R-induced lytic reactivation similarly to ΔNp63α, indicating that TAp63α may also be an inhibitor of lytic reactivation. ΔNp63ɑ and TAp63α are identical at the C-terminal domains, which contain sterile alpha motif (SAM) and the post-sterile alpha motif domains, features that ΔNp63β lacks. SAM motifs in p63α isoforms provide docking sites for proteins containing SAM motifs, indicating that a protein-protein interaction could be mediating ΔNp63ɑ and TAp63α lytic repression [82].

### TAp63α acts as a repressor of EBV lytic reactivation in the Akata Burkitt lymphoma cell line

**Fig 8:**
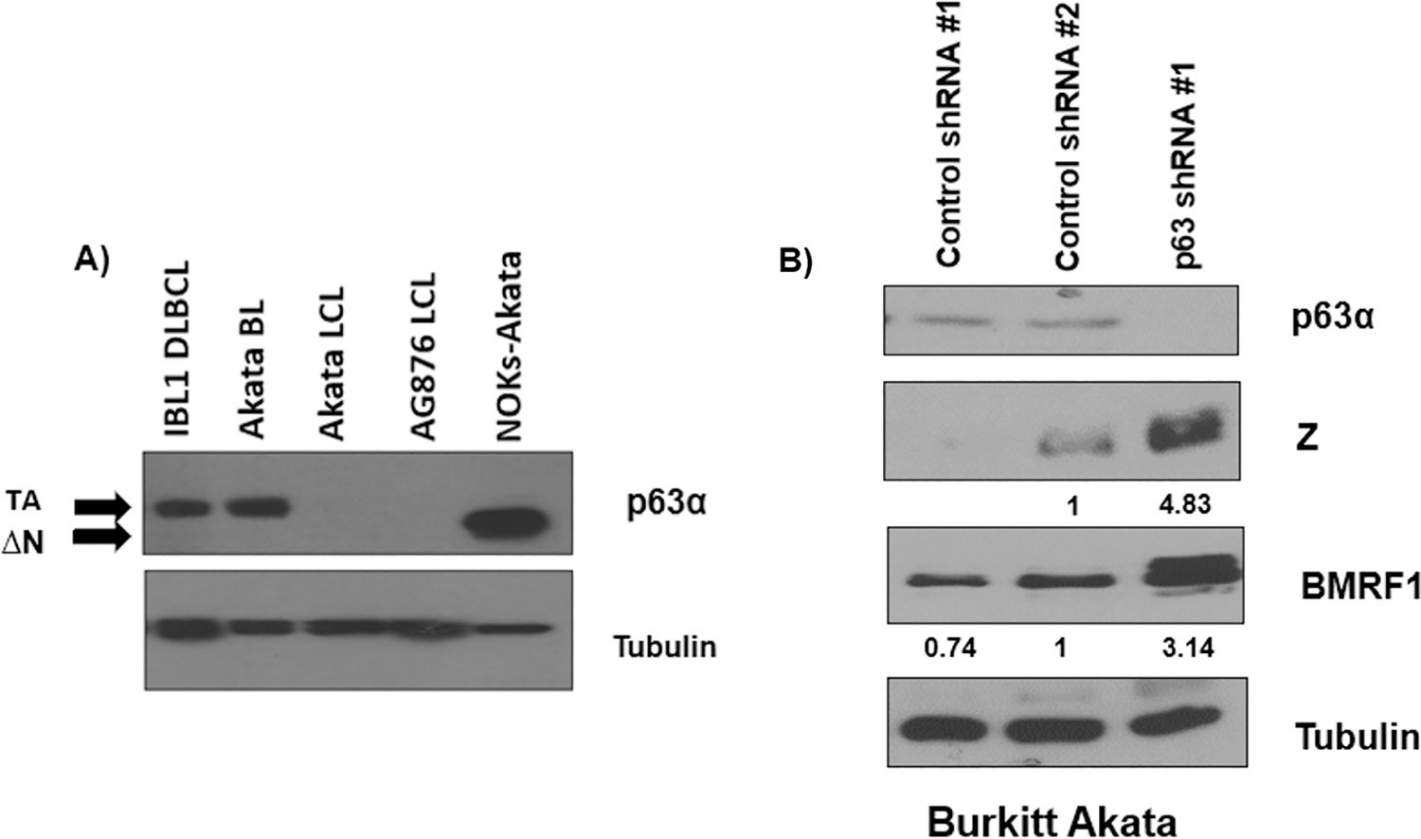
The TAp63α isoform of p63 inhibits EBV lytic reactivation in Akata Burkitt lymphoma cells. **A)** The level of TAp63α isoform expression in various EBV-infected B-cell lines were examined by immunoblot analysis; NOKs-Akata epithelial cells were used as a control for expression of the shorter ΔNp63α isoform. **B)** Akata Burkitt lymphoma cells were infected with two different lentiviral vectors expressing control shRNAs or a lentivirus vector expressing shRNA against p63, selected for puromycin resistance, and then assessed by immunoblot analysis for expression levels of the Z, R, BMRF1, TAp63α and tubulin proteins. The quantification of Z and BMRF1 protein levels was determined through ImageJ, normalized to tubulin, with the control #2 condition set as 1. These experiments are representative of two independent biological replicates.

Since TAp63α is expressed in some Burkitt lymphomas and diffuse large B cell lymphomas [79,80] and TAp63α over-expression inhibits lytic reactivation in NOKs-Akata cells, we asked whether TAp63α helps maintain EBV latency in a lymphoma cell line that expresses TAp63α. The level of TAp63α protein expression in various EBV-infected lymphoma cell lines or lymphoblastoid cell lines (including the ILB1 DLBCL line, the Akata BL cell line, an Akata EBV strain virus transformed lymphoblastoid cell line (LCL), and an AG876 EBV strain virus transformed LCL) was examined by immunoblot analysis. We found that the ILB1 DLCBL and Akata BL cell lines express TAp63α, which has a higher molecular weight compared to the ΔNp63α protein in the control NOKs-Akata epithelial cell sample (**Fig 8A**).

To determine if TAp63α represses EBV lytic reactivation in the B cell environment, we knocked down TAp63α expression in the Burkitt Akata cell line using an shRNA against p63 or infected the cells with two separate control shRNAs. EBV lytic protein expression was increased in cells expressing the shRNAs against TAp63α compared to the control conditions (**Fig 8B**). These results suggest that expression of TAp63α, like ΔNp63α, inhibits EBV lytic reactivation in EBV-infected human lymphoma cells and thus contributes to maintenance of EBV latency in this cell type.

### ΔNp63α inhibits constitutive Zp activity in AGS gastric cells and prevents KLF4-mediated activation of Zp

**Fig 9:**
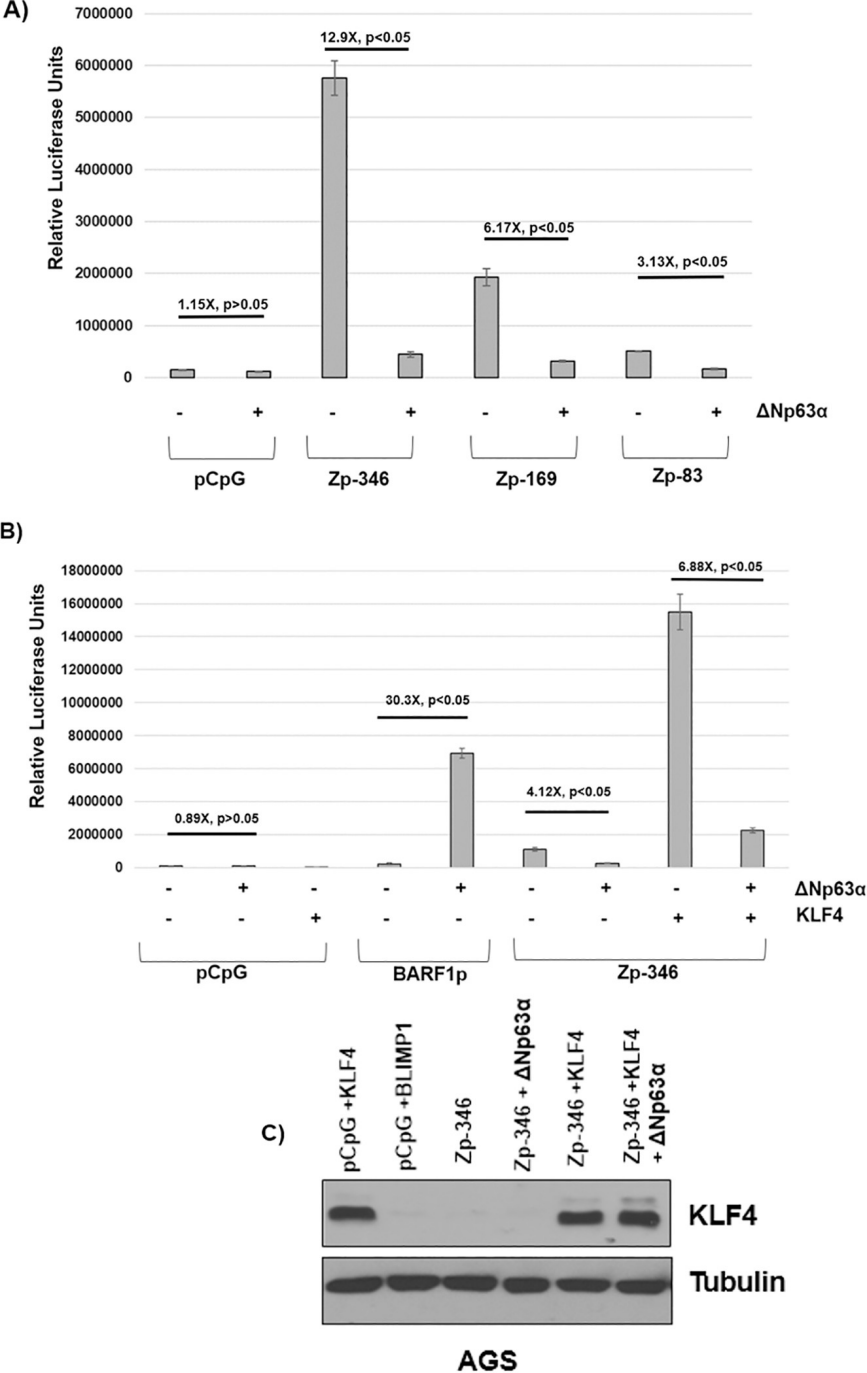
ΔNp63α inhibits Z promoter activity in reporter gene assays. **A)** Luciferase vectors containing various amounts of the Z promoter sequence, or the pCpG (promoterless) negative control vector, were transfected into EBV-negative AGS gastric carcinoma cells with or without an ΔNp63α expression vector. Luciferase activity was measured two days later. The average fold change in luciferase activity in ΔNp63α transfected cells versus control vector transfected cells for each promoter construct is shown, with error bars indicating standard error. Analysis of statistical significance between ΔNp63α and control conditions in the pCpG vector, Zp-346 vector, Zp-169 vector, and Zp-83 vector was performed using a two-sample t-test as indicated. **B)** AGS cells were transfected with the pCpG negative control luciferase vector, or luciferase vectors driven by the EBV early lytic BARF1 promoter, or the Z (Zp-346) promoter, in the presence or absence of a KLF4 expression vector, with or without co-transfected ΔNp63α, as indicated. The average fold change in luciferase activity for each condition (relative to each promoter construct transfected with control vector) is shown, as well as the standard error. Analysis of statistical significance between ΔNp63α and the control or KLF4 conditions in the pCpG vector, BARF1p vector, and Zp-346 vector was performed using a two-sample t-test as indicated. **C)** Immunoblot analysis of the Zp-luciferase assay samples shown in Fig 9B above was performed to assess KLF4 and tubulin levels. These luciferase assays are each representative of two independent replicates. A two-sample t-test was done to determine significance and the error bars indicate standard error of the mean.

Since R activates the Zp through indirect mechanism(s) involving poorly defined cellular transcription factors [25], we next asked if the constitutive activity of Zp in the gastric AGS cell line is also inhibited by ΔNp63α. EBV-negative AGS cells were chosen for these studies since Zp activity is very high in these cells (in contrast to most cell lines) due to high expression of positive regulators of the Z promoter such as c-jun, and a lack of negative regulators such as ZEB1/2 [83]. Luciferase reporter constructs containing either no promoter, or various portions of the Z promoter, were transfected into AGS cells with or without a ΔNp63α expression vector, and the amount of luciferase activity was quantified two days later. As shown in **Fig 9A**, ΔNp63α decreased the activity of each of the Zp reporter constructs tested, including a construct containing only 83 base pairs upstream of the BZLF1 transcription initiation site. In contrast, ΔNp63α did not repress activity of the promoterless luciferase vector, and, as previously reported [84], increased the activity of the EBV BARF1 promoter in the same reporter vector (**Fig 9B**).

We also examined the effect of **Δ**Np63α on the ability of co-transfected KLF4 to activate Zp in reporter gene assays. KLF4 is thought to activate Zp through a direct binding mechanism [16]. **Δ**Np63α decreased the ability of co-transfected KLF4 to activate Zp-driven luciferase activity but did not affect the total level of transfected KLF4 protein (**Fig 9B and 9C**). Together, these results strongly suggest that **Δ**Np63α inhibits Z promoter activation by positively acting regulators, including the EBV R protein and KLF4.

### Lytic repressor c-myc is increased by ΔNp63α

**Fig 10:**
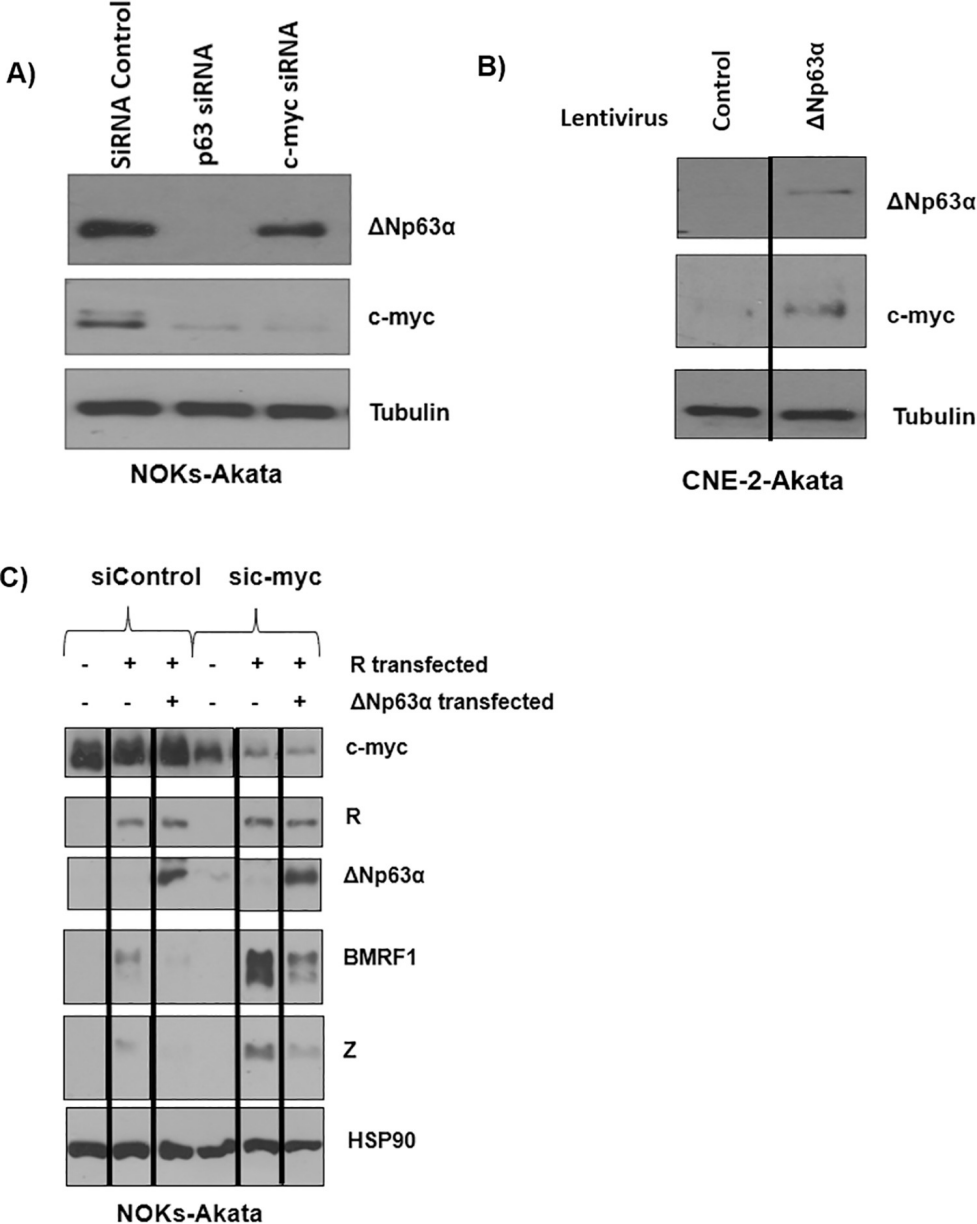
ΔNp63α expression increases the expression of the lytic repressor c-myc, but c-myc is not required for ΔNp63α mediated repression of lytic reactivation. **A)** NOKs-Akata cells were transfected with either ΔNp63α or c-myc targeting siRNAs or a control nonspecific siRNA. Two days after siRNA transfection, the cells were harvested for an immunoblot to determine the expression levels of ΔNp63α, c-myc, and tubulin (as a loading control). **B)** CNE-2-Akata cells were infected with a ΔNp63α expressing lentivirus or empty control. After five days of puromycin selection, the cells were harvested and an immunoblot performed to assess expression of the ΔNp63α, c-myc and tubulin proteins. **C)** NOKs-Akata cells were transfected with siRNAs against c-myc or a control siRNA for two days, and then transfected with or without an R expression vector in the presence or absence of ΔNp63α. Immunoblot was performed one day later to examine expression of c-myc, ΔNp63α, R, BMRF1, Z, and tubulin proteins. Black lines indicate where irrelevant lanes in the blot were removed; the original western blots used to construct this figure are shown in supplemental Fig 8. All experiments are representative of two independent experiments.

Since our results are consistent with a model in which ΔNp63α indirectly inhibits the activity of the Z promoter by upregulating expression of a cellular protein that inhibits Zp activity, we examined the effect of ΔNp63α over-expression, or ΔNp63α knock-down, on the level of c-myc in NOKs-Akata or CNE-2-Akata cells. C-myc was recently shown to be a potent inhibitor of EBV reactivation via repressive effects on Zp activity [19]. We observed that knockdown of ΔNp63α expression is associated with a concomitant decrease in c-myc expression **(Fig 10A)**, while over-expression of ΔNp63α increases c-myc expression **(Fig 10B),** consistent with previous reports that Np63α helps maintain c-myc expression in basal epithelial cells [19,85].

To determine if this alteration in c-myc expression is sufficient to completely explain the inhibitory effect of ΔNp63α on lytic reactivation, we knocked down c-myc expression using siRNAs in NOKs-Akata cells, and then transfected cells with an R expression vector in the presence or absence of a ΔNp63α expression vector. As shown in **Fig 10C**, although we confirmed that knock-down of c-myc alone does increase R-mediated lytic reactivation, we also found that ΔNp63α still inhibits R-mediated lytic reactivation even when c-myc expression is knocked down. Similar results were obtained in a second experiment (**S8 Fig**). Thus, while alterations in c-myc expression may well contribute to the ΔNp63α inhibitory effect, additional inhibitory mechanisms are also likely involved.

### ΔNp63α inhibits p38 MAPK activity, which also contributes to ΔNp63α suppression of lytic EBV reactivation

**Fig 11:**
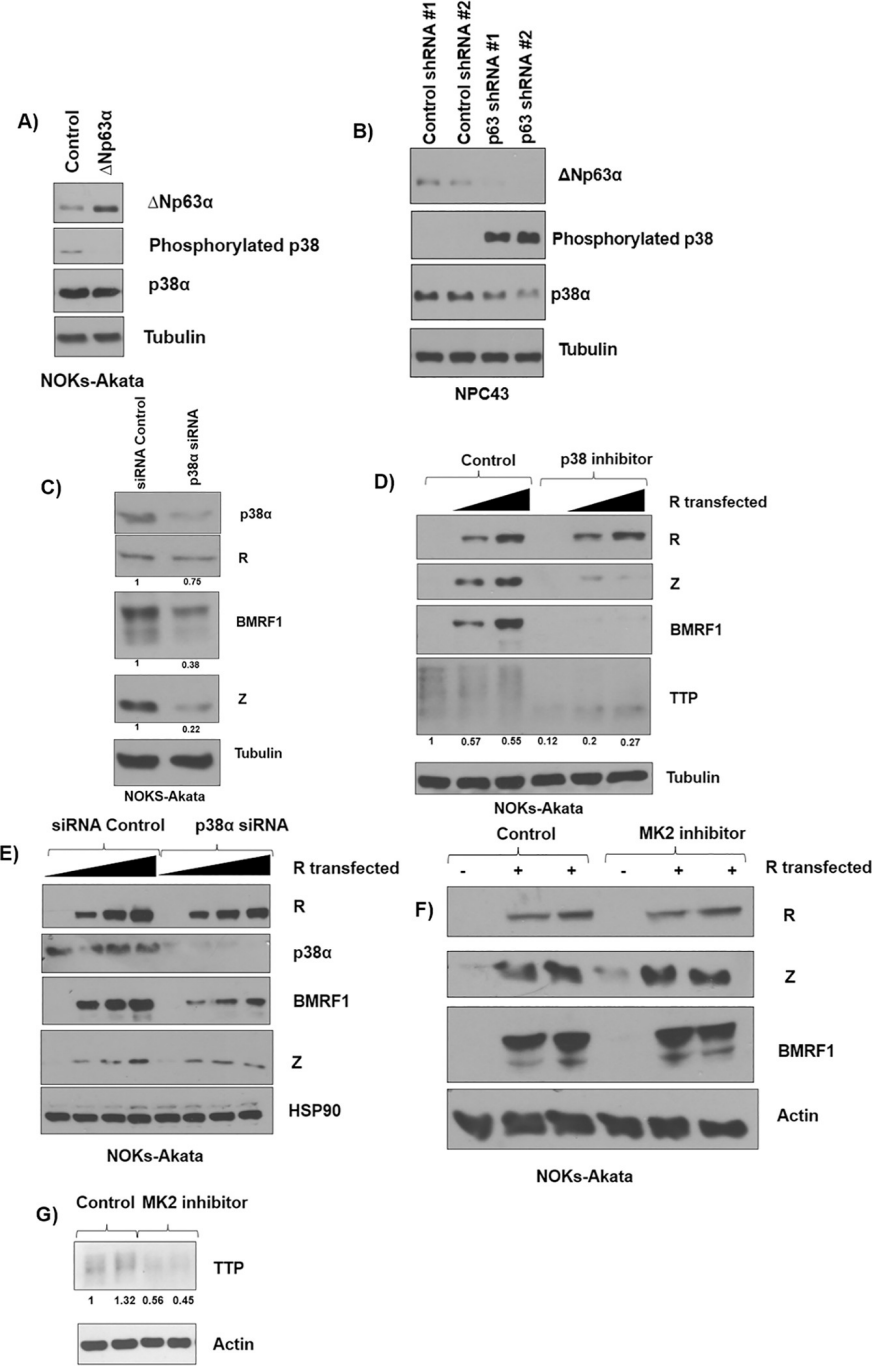
ΔNp63α over-expression decreases p38 kinase activity, which is required for R mediated lytic reactivation. **A)** Immunoblot analysis was performed on protein lysates harvested from NOKs-Akata cells infected with a lentivirus vector expressing ΔNp63α, or a control lentivirus vector, and expression of ΔNp63α, phosphorylated (activated) p38, total p38α (MAPK14), and tubulin (a loading control) was assessed. **B)** NPC43 cells were infected with a lentivirus expressing shRNA targeting p63 or a control sequence, then harvested for western blots to examine the expression of ΔNp63α, phosphorylated p38, total p38α and tubulin. Note that these samples were also used in Fig 2B. **C)** NOKs-Akata cells were transfected with either a control siRNA or an siRNA targeting p38α (MAPK14), and two days later immunoblot analysis was performed to examine expression of p38α (MAPK14), R, BMRF1, Z, and tubulin as indicated. Quantitation of R, BMRF1 and Z expression was determined through ImageJ, normalized to tubulin, and the siRNA control sample was set as 1. **D)** NOKs-Akata cells were transfected with or without an R expression vector in the presence or absence of 10 μM p38 MAPK inhibitor SB202190. Immunoblot analysis was performed two days later to examine expression levels of Z, R, BMRF1, TTP and tubulin as indicated. Expression of TTP was quantitated with ImageJ, normalized to tubulin with the first lane set as 1. **E)** NOKs-Akata cells were transfected with either a control siRNA or an siRNA targeting p38α (MAPK14) and then transfected with or without an R expression vector one day later. Immunoblots were performed one day after R transfection to examine expression of p38α (MAPK14), Z, R, BMRF1, and tubulin. **F)** NOKs-Akata cells were transfected with or without an R expression vector, in the presence or absence of 10 μM MK2 (MAPKAPK2) inhibitor (PF-364402 hydrate). Two days later immunoblot analysis was performed to examine expression of the Z, R, BMRF1, and actin proteins as indicated. **G)** NOKs-Akata cells treated with 10 μM MK2 (MAPKAPK2) inhibitor (same extracts used in Fig 11E) were also examined for expression of TTP (ZFP36) and tubulin as indicated. Quantitation of TTP expression was determined through ImageJ, normalized to tubulin, and the first control sample was set as 1. All results in this figure represent a minimum of two biological replicates.

ΔNp63α was recently reported to inhibit activity of cellular p38 MAP kinase by increasing expression of the cellular DUSP6 phosphatase [86]. Since ΔNp63α inhibits the ability of transfected R, but not Z, to induce lytic reactivation in epithelial cells (**Fig 4**), and we previously showed that the cellular p38 MAP kinase is required for R-mediated (but not Z-mediated) activation of the BZLF1 and BMRF1 lytic promoters in latently infected epithelial cells [25], we next asked whether ΔNp63α at least partially inhibits R-mediated activation of BZLF1 and BMRF1 expression by decreasing p38 kinase activity. To examine this possibility, we over-expressed ΔNp63α in NOKs-Akata cells (using a lentivirus vector) and examined the effect on p38 kinase activity. We confirmed that phosphorylated (activated) p38 is substantially decreased in ΔNp63α over-expressing NOKs-Akata cells compared to control cells (**Fig 11A**). Additionally, when we knocked down ΔNp63α in the context of the authentic NPC cell line NPC43, we found that this depletion increased phosphorylated p38 (**Fig 11B**).

Cellular p38 kinase is encoded by four different highly homologous cellular genes (MAPK 11–14). As shown in **Fig 11C,** knock-down of MAPK14 (p38α) expression (the most highly transcribed p38 encoding gene in NOKs-Akata cells [35]) inhibited constitutive Z and BMRF1 expression in NOKS-Akata cells, while R expression was only slightly affected. To examine how loss of p38 kinase activity affects the ability of R to induce various different lytic EBV promoters, NOKs-Akata cells were transfected with R in the presence or absence of a p38 kinase inhibitor (SB 202190, which affects both MAPK11 and MAPK14 p38 kinase activity), or an siRNA directed against the MAPK14 p38α kinase. As shown in **Fig 11D**, similar to the effect of ΔNp63α, treatment of cells with a p38 inhibitor greatly decreased R’s ability to activate expression of both the BZLF1 IE protein and BMRF1 early lytic proteins. As expected, inhibition of p38 kinase activity decreased expression of its known downstream target, TTP (ZFP36)[87]. Specific knock-down of MAPK14 (p38α) expression inhibited R’s ability to activate BMRF1 expression while having a lesser effect on BZLF1 expression **(Fig 11E**). Interestingly, the finding that specific knock-down of the p38α form of p38 kinase affected BZLF1 expression less than the p38 kinase inhibitor suggests that additional p38 kinase protein(s) may contribute to inhibition of BZLF1 expression, although this effect could also be due to incomplete knockdown. Together, these results reveal that p38 kinase activity is crucial for R-mediated lytic viral reactivation in NOKs-Akata cells and suggest that ΔNp63α inhibition of p38 kinase activity is an important mechanism by which it inhibits lytic reactivation.

### P38 kinase activation of lytic EBV infection does not require activation of the MK2 pathway

Although we previously showed that one mechanism by which p38 MAP kinase enhances lytic EBV reactivation is via phosphorylation and activation of the cellular ATF2 transcription factor (which directly binds to and activates the BZLF1 (Zp) promoter)[25], growing evidence suggests that p38 kinase commonly activates gene expression not only by enhancing transcription factor activity, but also by increasing the stability of ARE-containing RNA transcripts [87]. Since the major mechanism by which the p38 kinase promotes RNA stability is through phosphorylation and activation of the MK2 kinase (MAPKAPK2) [88], we examined the ability of a MK2 inhibitor to affect R-mediated lytic reactivation in NOKs-Akata cells. As shown in **Fig 11F**, inhibition of MK2 activity using a chemical inhibitor did not suppress the ability of transfected R protein to induce BZLF1 or BMRF1 expression. Loss of TTP (ZFP36) expression (which requires MK2 activity for high level expression [87]) in this experiment confirmed that MK2 activity was decreased (**Fig 11G**) [87].

### ΔNp63α over-expression during cis-platinum treatment of CNE-2-Akata and NOKs-Akata cells inhibits EBV lytic reactivation

**Fig 12:**
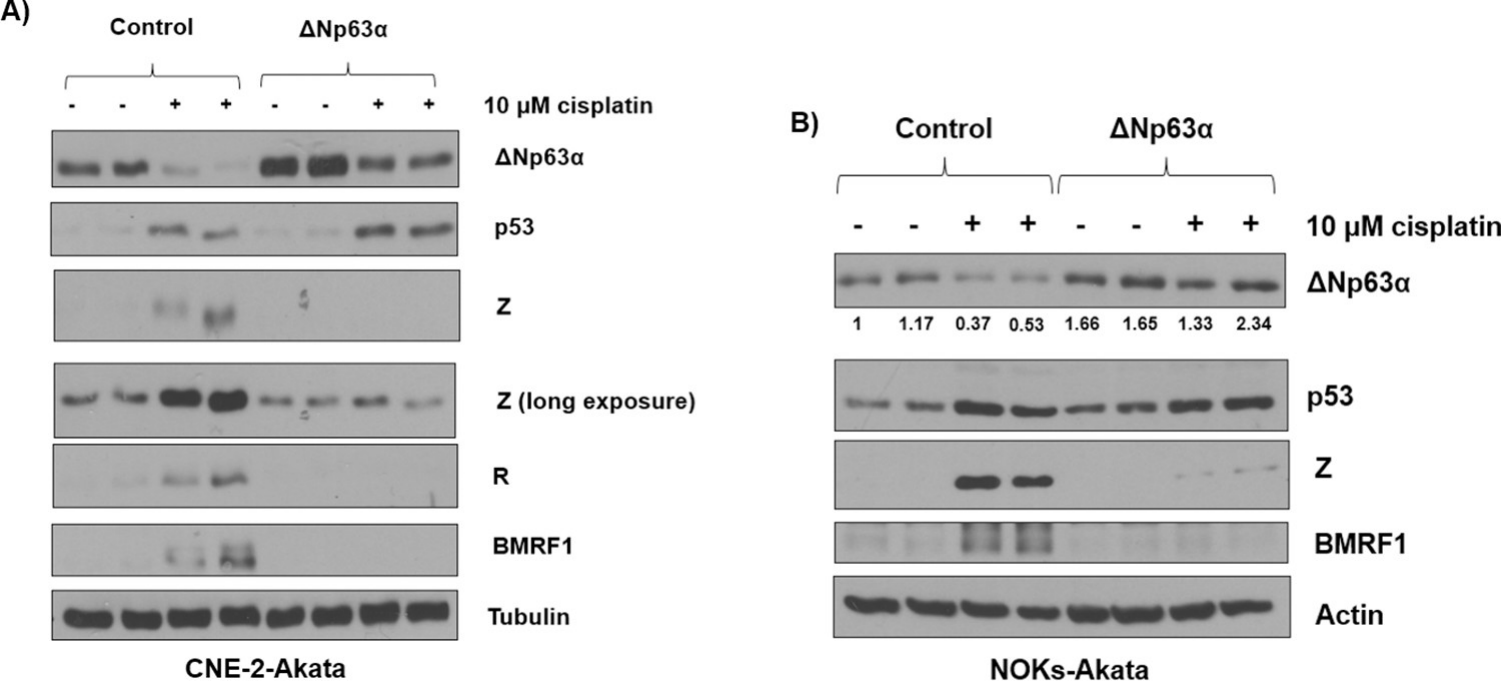
Cisplatin mediated lytic reactivation is curtailed during ΔNp63α over-expression. **A)** CNE-2-Akata cells infected with a lentivirus expressing ΔNp63α, or a control lentivirus, were treated with or without 10 μM cis-platinum and then examined by immunoblot analysis two days later for expression of p63, p53, Z, R, BMRF1, and the loading control tubulin. **B)** NOKs-Akata cells infected with a lentivirus expressing ΔNp63α, or a control lentivirus, were treated with or without 10 μM cis-platinum and then examined by immunoblot analysis two days later for expression of ΔNp63α, p63, p53, Z, BMRF1, and the loading control actin. The expression of ΔNp63α was quantified using ImageJ, normalized to tubulin, with the first control treated sample set as 1. All results in this figure have been independently confirmed four times.

Chemotherapy agents such as cis-platinum have been shown to potently induce EBV lytic reactivation [71,89,90], and the treatment of cells with cis-platinum is known to degrade ΔNp63α [91]. We hypothesized that ΔNp63α over-expression may therefore inhibit cis-platinum-induced lytic reactivation in epithelial cells. To examine this, we over-expressed ΔNp63α in CNE-2-Akata cells and then treated with 10 μM cis-platinum for two days to induce lytic reactivation. Over-expression of ΔNp63α resulted in a striking complete loss of lytic induction in response to cis-platinum (**Fig 12A**); similar results were obtained in NOKS-Akata cells (**Fig 12B**). These results indicate that ΔNp63α is a major repressor of cis-platinum-induced lytic reactivation and conversely, loss of intact ΔNp63α protein expression contributes to the lytic inducing effect of cis-platinum.

## Discussion

Infection of oropharyngeal epithelial cells plays an essential role in the EBV life cycle, particularly since these cells are the major source of transmissible virus. However, while EBV infection of normal oropharyngeal epithelial cells is highly lytic, and likely confined to the more differentiated cell layers, EBV infection of nasopharyngeal carcinoma cells is largely latent. Furthermore, the ability of NPC tumor cells to maintain EBV latency is likely required for their growth and survival. A major difference between NPC cells and the non-transformed oropharyngeal epithelial cells normally infected by EBV is that NPC cells are undifferentiated basal cells that express high levels of the basal cell transcription factor, ΔNp63α, which is lost when stratified epithelial cells differentiate. Here we demonstrate that both the basal epithelial cell specific ΔNp63α transcription factor, and the related isoform TAp63α (expressed in some EBV-positive lymphomas), act as potent negative regulators of EBV lytic reactivation. These findings help to explain how EBV can achieve latent infection in undifferentiated NPC and B cell lymphomas.

EBV has two different IE proteins, Z and R, which encode viral transcription factors that cooperate to induce lytic reactivation of EBV. Our studies here show that the major inhibitory effect of ΔNp63α on lytic EBV reactivation occurs via negative regulation of the BZLF1 gene promoter (Zp). We found that ΔNp63α prevents the ability of transfected R protein to induce Z protein expression in latently infected epithelial cells, while not inhibiting the ability of transfected Z protein to induce R expression and downstream early lytic EBV proteins (**Fig 4**). These results indicate that Z over-expression (under the control of a heterologous promoter) is sufficient to bypass the inhibitory effect of ΔNp63α. Interestingly, in contrast to the effect on R-mediated early lytic BMRF1 protein expression, we found that ΔNp63α does not inhibit R-mediated activation of the early lytic SM protein (**Fig 4D**). This difference likely reflects the fact that the SM lytic gene can be turned on by R alone (even in the absence of Z expression [67,92]), while activation of BMRF1 gene expression requires both the Z and R proteins [29,65,66]. Our finding that ΔNp63α also inhibits late lytic protein expression in R-transfected cells reflects the known requirement for Z to complete the lytic form of viral DNA replication [29,33] and to initiate subsequent late viral gene expression. Thus, loss of sufficient Z expression is the key defect impeding lytic viral reactivation in ΔNp63α expressing cells.

Although ΔNp63α is an essential regulator of epithelial cell differentiation and is well known to inhibit differentiation of epithelial cells by repressing expression of key cellular transcription factors such as KLF4 [61] that are required for epithelial cell differentiation, we found here that ΔNp63α inhibits Zp activity even in cells that cannot differentiate such as AGS cells and CNE-2 cells (**Figs 4A, 4D and 9**). Thus, although EBV uses differentiation-dependent cellular transcription factors such as KLF4 and BLIMP1 to ensure that Zp and Rp are both activated when EBV-infected epithelial cells differentiate [15,16], it has usurped the functions of the basal cell specific transcription factor ΔNp63α to inhibit Zp activity even in the absence of KLF4 and BLIMP1. We show here that ΔNp63α inhibits the constitutive activity, as well as KLF4-induced activity, of Zp-driven reporter gene constructs in EBV-negative AGS gastric carcinoma cells (**Fig 9**).

EBV-negative AGS cells do not normally express ΔNp63α and in contrast to most other cell types are remarkable for their high level of constitutive Zp activity [83]. We found that ΔNp63α strongly inhibits constitutive Zp activity in AGS cells and this effect was observed even with the smallest Z promoter construct studied (which contains only 83 base pairs upstream of the transcriptional initiation site). Of note, each of the Zp luciferase constructs used in this study contains the AP-1-like “ZII” motif, which binds to the AP-1 and ATF2 [25] transcription factors and is required for constitutive Zp activity. ΔNp63α also inhibits the ability of transfected KLF4 protein to enhance Zp activity in AGS cells, without affecting the level of transfected KLF4 (**Fig 9**). We previously showed that KLF4 binds directly to Zp and contributes to differentiation-dependent lytic EBV reactivation in epithelial cells [16]. Together, these results suggest that ΔNp63α inhibits the ability of multiple different stimuli to induce Zp activity.

To examine whether ΔNp63α inhibits lytic EBV reactivation by binding directly to the Zp or Rp IE promoters, we performed ChIP assays using a FLAG-tagged ΔNp63α expression vector (**Fig 6A**). Although we confirmed that ΔNp63α bound to a positive control cellular promoter in these assays, we did not observe binding of ΔNp63α to the Zp or Rp EBV IE promoters. To determine whether ΔNp63α blocks R binding to known RRE sites in the early lytic BMRF1 and SM promoters, we also performed ChIP assays using a FLAG-tagged R expression vector to compare R binding to these promoters in the presence and absence of co-transfected ΔNp63α (**Fig 6B**). ΔNp63α co-transfection did not affect R binding to either the BMRF1 or SM promoters. Together, these results suggest that the inhibitory effect(s) of ΔNp63α on Zp activity do not require either direct ΔNp63α binding to the Zp or Rp IE promoters, or inhibition of R binding to other EBV promoters.

To further examine potential mechanism(s) for the inhibitory effect of ΔNp63α on Zp activity, we compared the ability of different p63 isoforms to block R-mediated lytic EBV reactivation in NOKs-Akata cells (**Fig 7**). These studies revealed that the C-terminal domain of ΔNp63α is required for the inhibitory effect. Since the TAp63α isoform (which is expressed in some EBV+ lymphomas) shares this inhibitory C-terminal domain, we also asked whether this isoform can block lytic viral reactivation. Importantly, we found that the Burkitt Akata lymphoma line expresses TAp63α, and that knockdown of TAp63α in these cells increases constitutive lytic EBV protein expression (**Fig 8**). Thus, other p63 family members may also play a role in maintaining EBV latency in some EBV-infected lymphomas.

Our results here suggest that one mechanism by which ΔNp63α promotes viral latency is by increasing expression of c-myc (**Fig 10**). ΔNp63α is known to enhance c-myc expression in basal epithelial cells [93], and c-myc was recently shown to potently inhibit Z expression and lytic reactivation in EBV-infected cells [19]. We confirmed that ΔNp63α activates c-myc expression in NOKs-Akata cells and showed that siRNA-mediated knock-down of c-myc increases the ability of transfected R protein to induce Z and BMRF1 expression (**Fig 10**). Nevertheless, since we found that ΔNp63α still inhibits R-mediated lytic reactivation in NOKs-Akata cells even when c-myc expression is greatly decreased using siRNA (**Fig 10**), the ΔNp63α effect on c-myc expression is unlikely to be the only mechanism by which ΔNp63α promotes viral latency.

Instead, our experiments here suggest that another major mechanism by which ΔNp63α promotes viral latency in epithelial cells is by decreasing p38 kinase activity. We found that ΔNp63α expression inhibits p38 kinase activity in NOKs-Akata cells, and that inhibition of p38 kinase activity reduces both constitutive, and R-mediated activation of the Z and BMRF1 proteins in NOKs-Akata cells (**Fig 11**). Although the mechanism(s) by which R increases Zp activity in the context of the intact viral genome are not completely understood, direct DNA binding of R to the Z promoter does not appear to be involved, and instead R activation of Zp is thought to be mediated through indirect effects of R on cellular transcription factors [28,63,64,71]. We previously showed that inhibition of p38 kinase activity blocks the ability of over-expressed R protein, but not over-expressed Z protein, to induce lytic EBV reactivation in latently infected epithelial cells, and correlated this effect with the ability of p38-phosphorylated ATF2 transcription factor to bind to and activate the BZLF1 promoter [25]. More recently, the ability of the late lytic BGLF2 tegument protein to promote lytic reactivation was also shown to be at least partially mediated through p38 kinase activation [94,95].

In addition to its requirement for R-mediated and BGLF2-mediated lytic EBV reactivation in epithelial cells, a growing literature indicates that p38 kinase activity is required for the ability of multiple different types of stimuli to induce lytic EBV reactivation in both epithelial cells and B cells. Indeed, p38 kinase inhibitors have been shown to block viral reactivation in response to such diverse stimuli as B-cell receptor activation, chemotherapy agents, phorbol esters, reactive oxygen species, and TGF-β [96–101]. Given the large number of direct p38 kinase substrates, and the multiple downstream pathways that are indirectly affected by this kinase, we speculate that p38 kinase contributes to lytic EBV reactivation through multiple different mechanisms. Our results here suggest that one of the primary effects of this kinase on EBV reactivation is to enhance BZLF1 promoter activation in response to both viral and cellular transcription factors. Interestingly, a number of different cellular transcription factors that have previously been shown to bind to and activate the BZLF1 promoter (including ATF1, ATF2, c-jun, c-fos, XBP1, HIF-1α, and p53) are known to be directly phosphorylated and activated by p38 kinase [102]. Furthermore, p38 kinase more globally enhances transcription by activating downstream kinases such as MSK1 and MSK4 that directly phosphorylate histone sites such as H3 S28; this phosphorylation then reduces inhibitory modifications on H3 K27 that recruit the polycomb complex [103–105]. Of note, p38γ activity has previously been shown to increase during epithelial cell differentiation and to be required for efficient induction of differentiation genes such as involucrin [106].

In addition, p38 kinase activity is increasingly recognized to exert many of its cellular effects by increasing stability of a subset of cellular RNAs [88]. This effect is mediated largely through activation of the MK2/MKK3 kinases, which phosphorylate and inactivate a cellular protein, ZFP36 (TTP), that induces degradation of RNAs containing AU-rich 3’ LTRs [87]. However, treatment of NOKS-Akata with an MK2 inhibitor did not impact R-induced lytic reactivation, indicating that p38 may facilitate lytic reactivation through pathways independent of effects on RNA stability (**Fig 11F**). Interestingly, p38 kinase was also recently reported to inhibit nonsense mediated decay (NMD) of RNA in cells with DNA damage [107]. Since NMD was recently shown to promote degradation of the transcript encoding the BZLF1 and BRLF1 genes in latently infected cells [108], decreasing NMD of this transcript in the context of the intact viral genome could be another post-transcriptional mechanism by which p38 kinase promotes lytic EBV reactivation.

Finally, we show here that the recently described ability of the chemotherapy agent cis-platinum to induce ΔNp63α degradation in epithelial cells [91] can potentially be used to potentiate “lytic induction” therapy for EBV-positive epithelial tumors. Lytic induction therapy of latently infected EBV-positive tumor seeks to identify small molecules that can reactivate the lytic form of viral infection in tumors, thereby using the virus itself to help kill the tumor cell. Interestingly, we previously showed that p38 kinase activity is required for chemotherapy to induce lytic EBV reactivation in certain epithelial cell lines [89]. Here we confirmed that cis-platinum treatment of EBV-infected epithelial cells does, indeed, result in loss of intact ΔNp63ɑ expression and showed that restoration of ΔNp63ɑ expression in cis-platinum treated cells reduces the amount of lytic EBV reactivation (**Fig 12A and 12B**). Thus, the use of chemotherapy drugs such as cis-platinum that can not only induce DNA damage and p53/ATM activation (known activators of lytic EBV reactivation [71,73]), but can simultaneously degrade ΔNp63ɑ, is predicted to be the most effective approach for achieving efficient lytic induction therapy in EBV-positive NPC tumors.

## Materials and methods

### Cell culture

The normal oral keratinocytes (NOKs) cell line (a generous gift from Karl Munger of Tufts University via Paul Lambert of the University of Wisconsin) is a telomerase-immortalized normal oral keratinocyte cell line, grown in keratinocyte serum-free media supplemented with 12.5 mg bovine pituitary extract, and 0.1 μg epidermal growth factor per 500ml of media (KSFM, Lifetech). NOKs were derived as previously described [109]. p53KO NOKs were derived by CRISPR-Cas9 mutagenesis of the p53 gene as previously described [73]. In brief, these NOKs- cells were infected with a lentivirus containing a guide RNA (5′-TCGACGCTAGGATCTGACTG-3′) targeting the second exon of p53 and selected with puromycin. EBV-infected NOKs-Akata cells were created as previously described [29] and were maintained with 50 μg/ml G418 antibiotic selection in addition to the media/growth supplements used to grow NOKs cells. The CNE-2 cell line was derived initially from NPC tumors but has been subsequently shown to have HeLa cell genome contamination and HPV infection [110,111]. CNE-2-Akata cells (a generous gift from K.W. Lo of the Chinese University of Hong Kong via Diane Hayward) are stably EBV-infected with the Akata strain of EBV (containing a G418 resistance gene cassette and GFP gene inserted into the EBV BXLF1 gene) and were grown in DMEM with 10% fetal bovine serum, 1% pen-strep, and 400 μg/ml G418 antibiotic selection. The NPC43 cell line is an authentic EBV-infected nasopharyngeal carcinoma cell line described previously [59] and was grown in RPMI media supplemented with 10% fetal bovine serum, 1% pen-strep, and 4 μM Y-27632. The AGS cell line is an EBV-negative gastric carcinoma cell line that was obtained from the ATCC and was grown in F12 media with 10% fetal bovine serum and 1% pen-strep. The AGS-Akata cell line is an AGS cell line stably infected with the Akata strain of EBV (containing a G418 resistance gene cassette and GFP gene inserted into the EBV BXLF1 gene) was derived as previously described [112] and was a kind gift from Lindsay Hutt-Fletcher. The AGS-Akata cell line was grown in F-12 media with 10% fetal bovine serum, 1% pen-strep, and 400 μg/ml G418 antibiotic selection. Both the uninfected and EBV-infected AGS were cured (in the Hutt-Fletcher lab) of the contaminating SV5 virus present in most AGS lines. The Burkitt lymphoma cell line Akata (Burkitt Akata) (a gift from Lindsay Hutt-Fletcher) was derived as previously described by super-infecting an EBV-negative Akata Burkitt lymphoma cell clone with the Akata strain of EBV (containing a G418 resistance gene cassette and GFP gene inserted into the EBV BXLF1 gene)[112] and was maintained with RPMI media with 10% fetal bovine serum, 1% pen-strep and 500 μg/ml G418 antibiotic selection. EBV-negative Akata cells were a kind gift from Kenzo Takada of Hokkaido University, Japan, via Bill Sugden of the University of Wisconsin and have been previously derived as described [113]. The IBL1 diffuse large B cell lymphoma line is a gift from Ethel Cesarman and was derived as previously described [114]. The AG876 and Akata lymphoblastoid cell lines (LCLs) were derived from peripheral blood B cells transformed with either the AG876 or Akata strains of EBV, respectively, as previously described [115] and were maintained with RPMI supplemented with 10% fetal bovine serum and 1% pen-strep.

### Collagen membrane differentiation

Collagen membrane differentiation was performed as previously described [116]. In brief, approximately 5x10^5^ total NOKs-Akata cells were seeded onto a collagen-treated transwell membrane (Corning #3460) with KSFM media on both the basal and apical surfaces of the membrane. After 24 hours, when cells were 100% confluent, apical media was removed and the basal media exchanged for Epilife media (Thermofisher # MEPICF500) supplemented with 10% FBS, 1.4 mM CaCl_2_, and 5 ug/ml ascorbic acid. Cell extracts on the collagen were harvested with sumo lysis buffer for immunoblot analysis after three days of differentiation.

### Immunoblots

Immunoblots were performed as previously described [117]. Briefly, cell lysates were harvested with sumo lysis buffer (1:3 mixture of buffer I (5% sodium dodecyl sulfate (SDS), 0.15 M Tris-HCl (pH 6.8), 30% glycerol) and buffer II (25 mM Tris-HCl (pH 8.3), 50 mM NaCl, 0.5% NP-40, 0.5% deoxycholate, 0.1% SDS) with protease inhibitors (cOmplete, Roche). Quantitation of protein concentration was conducted with a DC Bio-rad protein assay. The lysates were separated using a 10% polyacrylamide gel and then transferred onto a nitrocellulose membrane. The membranes were subsequently blocked with 5% milk consisting of 0.1% Tween 20 and 1X PBS for one hour. Membranes were then incubated with primary antibody overnight. The following day the antibodies were removed and the membrane was washed with wash buffer (1X PBS, 0.1% Tween 20) three times for 5 minutes. The membrane was then incubated with secondary antibody suspended in 5% milk for one hour, before washing with wash buffer three times for 10 minutes before treatment with ECL (Thermofisher) and imaging.

### Immunohistochemistry

Immunohistochemistry (IHC) was conducted as previously described [118]. To summarize, paraffin-embedded slides were initially heated on 70°C heat blocks and deparaffinized in xylene two times for 10 minutes. The slides were then hydrated in a series of alcohols (100%, 90%, 70%) and subsequently water for a period of 5 minutes each. The slides were then boiled in 1X sodium citrate buffer for 20 minutes and allowed to cool for an hour before blocking with horse serum. Primary antibodies were added to the slides for an hour, and then the primary was washed off in 1X PBS before adding secondary for thirty minutes. DAB was added for a period of approximately 15 seconds, and then horseradish peroxidase for approximately 10 seconds. Hematoxylin was used as a counterstain and the slides were dehydrated in alcohols before mounting. Z antibody (Santa Cruz, catalog # sc-53904) and p63α antibody (Cell Signaling Technologies catalog # 13109) were both used at a 1:200 dilution.

### Luciferase assays

Luciferase assays were conducted as previously described [16]. To summarize, 48 hours after transfection EBV-negative AGS cells were washed once with PBS and suspended in 200 μL of reporter lysis buffer (Promega). The lysate was flash-frozen once and pelleted by centrifugation. The supernatant of the lysate was used for the luciferase assays according to the manufacturer’s instruction using a BD Monolight 3010 luminometer (BD Biosciences).

### Chemicals

Cisplatin (Sigma, catalog#1134357) was made up at 10 mM and used at 10 μM. SB202190 p38 kinase inhibitor was purchased from Santa Cruz (sc-202334) was used at 10 μM. Phorbol 12-myristate 13-acetate (TPA) was purchased from Sigma (catalog #P8139) used at 20 ng/ml. MK2 inhibitor PF-364402 hydrate was purchased from Millipore-Sigma (catalog #PZ0188) and used at 10 μM. Y-27632 was purchased from R&D systems (catalog #1254/10) and used at 4 μM. Control conditions were treated equal amounts of the solvent.

### Antibodies

The following antibodies were used for immunoblot analyses in this study: anti-R rabbit polyclonal antibody directed against the R peptide (peptide sequence EDPDEETSSQAVKALREMAD 1: 2500), anti-p63ɑ (Cell Signaling Technologies catalog # 13109 1:1000), anti-ΔNp63 (Biolegend, catalog #619001), anti-KLF4 (Cell Signaling Technologies, catalog #4038 1: 1000), anti-BZLF1 (Santa Cruz, catalog # sc-53904, 1:500), anti-BMRF1 (Millipore, catalog # MAB8186, 1: 2500), anti-p18 (Thermo-Fisher Scientific, # PA1-73003, 1:2000), anti-BLIMP1 (Cell Signaling Technologies catalog # 9115S 1:1000), anti-involucrin (Sigma, catalog #19018, 1:3000), anti-c-myc (Abcam catalog# ab32072, 1:10000), p38 MAPK14 antibody (Cell Signaling Technologies catalog#9218 1:1000) and phospho-p38 MAPK (Cell Signaling Technologies catalog #9211 1:1000), anti-EBV SM polyclonal rabbit antibody (1:750), a generous gift from Sankar Swaminathan of the University of Utah, anti-Tristetraprolin (Cell Signaling Technologies catalog# #71632 1:1000). The secondary antibodies used were Horseradish peroxide (HRP)- labeled goat anti-mouse antibody (Thermo Scientific# 31430, 1:5000), HRP- labeled donkey anti-goat antibody (Santa Cruz#sc-2056, 1:5000), and HRP- labeled goat anti-rabbit antibody (Fisher Scientific 1:10000).

### Plasmids

All plasmid DNA was prepared using the Qiagen Maxi-prep kit according to the manufacturer’s instructions. The plasmid pSG5 was purchased from Stratagene. pSG5-R and pSG5-Z (kind gifts from Diane Hayward of John Hopkins University) contain the BZLF1 (Z) and BRLF1 (R) immediate-early genes driven by the SV40 promoter as previously described [14,119]. The pCpG, BARF1p, and the Zp luciferase expression vectors have been described previously [16,29]. ΔNp63ɑ-FLAG (Addgene # 26979, a gift from David Sidransky of John Hopkins University) encodes the ΔNp63ɑ gene with a FLAG tag. ΔNp63ɑ pReceiver-Lv105 (Genecopoeia product # Z57540-Lv105) is a ΔNp63ɑ lentiviral expression vector, and plenti is the lentiviral empty control (Addgene #39481, a gift from Ie-Ming Shih of John Hopkins University). ΔNp63ɑ-CMV was purchased from Genecopoeia (catalog #EX-Z5740-M02). pCMV3FC and BRLF1-pCMV3FC (containing a FLAG-tagged BRLF1 protein) are both kind and generous gifts from Lori Frappier of the University of Toronto [120]. Mouse ΔNp63ɑ-myc, TAp63ɑ-myc, and ΔNp63β-myc plasmids are generous gifts of Xiaohua Su at MD Anderson and Elsa Flores at the Moffitt Cancer Center.

### siRNAs

siRNAs against ΔNp63ɑ (sc-36161A, sc-36161B), c-myc (sc-29226, sc-44248), MAPK14 (sc-29433), and control siRNAs A and C (sc-37007, sc-44231) were purchased from Santa Cruz. siRNAs at 20 pM final concentration were transfected in 12 well plates using RNAiMAX (Invitrogen) according to the manufacturer’s protocol. After two days, the cells were harvested with sumo lysis buffer for immunoblot analysis.

### Transient transfections

DNA was transfected into NOKs-Akata, CNE-2-Akata, AGS-, and AGS-Akata using the Lipofectamine 2000 (Thermo Fisher #11668019) system according to the manufacturer’s protocol. Generally, 500 ng of total DNA with 1.5 μL Lipofectamine 2000 was used per condition to transfect epithelial cells that were approximately 70% confluent in a 12 well plate.

### shRNAs and lentivirus packaging

shRNAs against ΔNp63ɑ purchased from Horizon (catalog#RHS4533-EG8626). Lentivirus packaging was done as previously described [16]. Packaging components pCMV-VSV-G (Plasmid #8454, a gift from Bob Weinberg of the Massachusetts Institute of Technology) and pSPAX2 (Plasmid #12260, a gift from Didier Trono from the École Polytechnique Fédérale de Lausanne) were purchased from Addgene. To package the lentiviruses, 4 μg of the vector, 0.6 μg of VSV-G, and 1.4 μg of psPAX2 were transfected into 293T cells plated in a 10 cm dish. The cell’s media was changed 24 hours post-transfection and viral supernatant was harvested on days 2 and 3 to infect cells. Cells were selected with puromycin three days post-infection to derive a stable cell line.

### Organotypic rafting

Cells were stratified by organotypic rafting as described previously [16]. To briefly summarize, a dermal equivalent was created using transwell inserts (24 mm diameter, 0.4 μM pore Costar) that were coated with 1ml collagen mix (3 mg/ml Wako) also containing F-media, 10% FBS and 1% pen-strep. This layer was coated with an additional 2.5 ml collagen mix containing F-12 media, 10% FBS, 1% pen-strep, and 4.5 X 10^5^ early- passage human fibroblasts (EF-1-F). This dermal equivalent was suspended in F-12 medium supplemented with 10% FBS and 1% pen-strep. Four days later, 2.1 x 10^5^ NOKs-Akata cells were plated on the dermal equivalent suspended in keratinocyte plating media (F-medium [1.88 mM Ca2^+^]) supplemented with 0.5% FBS, adenine (24 μg/ml), cholera toxin (8.4 ng/ml), hydrocortisone (2.4 μg/ml), and insulin (5 μg/ml). The cells were allowed to grow for four days to reach confluence, and at this point the media was exchanged for cornification media (keratinocyte plating medium containing 5% FBS and 10 μM C_8:0_), and the cells were lifted to the air liquid interface so that only the basal cells were supplied with media. After 11 days of differentiation in cornification media that was replaced every other day, the cells were harvested and embedded in 2% agar-1% formalin, and fixed in 10% neutral buffered formalin overnight. The raft tissues were subsequently embedded in paraffin and sectioned in 4 μM cross sections.

### Chromatin immunoprecipitation (ChIP) and quantitative PCR

ChIP assays were performed as described previously [121]. For p63 ChIP assays, AGS-Akata cells (using three 10cm dishes per condition) were transfected with a pCDNA empty vector control or a p63-FLAG expression vector. For the R-FLAG ChIP assays, CNE-2-Akata cells were transfected with BRLF1-pCMV3FC (which contains a FLAG tag), BRLF1-pCMV3FC with ΔNp63ɑ-CMV, or pCDNA empty vector control. 24 hours post-transfection, the cells were fixed with 1% paraformaldehyde for 10 minutes and quenched with 125 mM glycine for 5 minutes. The cells were then pelleted by centrifugation at 1500 rpm for 10 minutes, washed with PBS. The cell pellets were then lysed in cell lysis buffer (10 mM Tris pH 8.0, 10 mM NaCl, 0.2% NP40) with protease inhibitors (cOmplete, Roche) to remove cytoplasmic protein. Nuclear pellets were lysed in nuclei lysis buffer (50 mM Tris-HC pH 8.0, 10 mM EDTA pH 8.0, 1% SDS) with protease inhibitors and the mixture was left on ice for 10 minutes. The nuclear lysates were then diluted in IP dilution buffer (20 mM Tris-HCl pH 8.0, 2 mM EDTA, 150 mM NaCl, 1% Triton X100, and 0.01% SDS) and sonicated 4 cycles (30 seconds ON at 10 watts/ 90 seconds OFF) (Fisher Scientific, Sonic Dismembrator Model 100). Approximately 25 μg DNA were diluted with IP dilution buffer, blocked with magnetic A/G beads (Thermo-Fisher, 88802) for one hour, and immunoprecipitated with M2 FLAG (Sigma-Aldrich, M8823-1 ml) beads overnight. The samples were then washed with low salt, high salt, and lithium chloride wash buffer for 15 minutes each, followed by two 15-minute washes with T_10_E buffer. Protein/chromatin complexes were eluted with elution buffer (0.1M NaHCO_3_, 1% SDS). Crosslinked protein DNA complexes were reverse cross-linked with 0.3M NaCl for 4 hours at 65°C with RNAse A, then proteinase K were added to all samples. Sample DNA was purified with phenol-chloroform, precipitated with EtOH, and resuspended in T_10_E for real-time PCR. Purified DNA was quantified using following primers (Z promoter: 5’ CCGGCAAGGTGCAATGTTTAG/ 3’ CATCACAGAGGAGGCTGGTG, R promoter: 5’ TGCCGGCTGACATGGATTACT/ 3’ GATGCTGATGCAGAGTCGCC, BMRF1 promoter: 5’ CACTGCGGTGGAGGTAGAG/ 3’ GGTGGTGTGCCATACAAGG, NECTIN1, 5’ TGAGCCTGTAGGACCAGAATCA/ 3’ TTTCCCACTCAAGCTGTGTCTCT) and iTaq universal SYBR green supermix (Bio-Rad) using a CFX96 touch real-time PCR detection system (Bio-Rad). Purified input DNAs were used in real-time PCR for standardization. The experiments shown are representative of two independent experiments and error bar indicating standard error of the mean within experiments.

### Viral titer assay

Media containing infectious virus was harvested from CNE-2-Akata cells three days after a transfection of either R, R with ΔNp63ɑ, or a vector control. The media was filtered with a 0.8 μM filter (Millipore-Sigma), and 10 μL of virus prep was used to infect EBV negative Akata Burkitt cells. 24 hours after infection, these cells were treated with 20 ng/ml of TPA, and 3 mM sodium butyrate. After an additional 24 hours, GFP-positive cells were counted to determine quantity of infectious virus. The experiment shown is the average of three independent viral titers, with an error bar indicating standard error of the mean, and two-sample T-test used to determine significance.

## Supporting information

S1 Fig The original western blots used to construct Fig 2A are shown. The lanes used in the blot shown in Fig 2A are labelled. Lanes not used in the final figure are indicated by a “I” symbol.
(TIF)

S2 Fig NOKs-Akata were transfected with siRNAs against ΔNp63α or a control sequence. Two days after transfection the cells were harvested for immunoblot analysis and the expression of ΔNp63α, Z, R, and tubulin was determined.
(TIF)

S3 Fig **A)** NOKs-Akata infected with either a lentivirus expressing ΔNp63α or a control lentivirus were differentiated on a collagen membrane or untreated for three days before harvesting for immunoblots examining the expression of ΔNp63α, R, Z, BMRF1, involucrin, BLIMP1, and tubulin. **B)** The original western blots used to construct supplemental Fig 3A are shown; the lanes used for the figure are labeled 1 through 4, and lanes not used are indicated by a “I” symbol.
(TIF)

S4 Fig The original western blots used to construct Fig 4A are shown in the right panel. The lanes used in the blot shown in Fig 4A are labelled 1–6 as indicated. Lanes not used in the final figure are indicated by a “I” symbol. Different western blots were used to derive the ΔNp63α and R expression levels whereas the same three western blots were used to derive the Z, BMRF1, and tubulin expression levels. “Blank” refers to lanes where no protein was loaded. Note that the same protein lysates were used in each of the western blots shown.
(TIF)

S5 Fig **A)** AGS-Akata cells were transfected with a vector control or Z expression vector in the presence of absence of ΔNp63α expression vector, as indicated. Western blots were performed to examine the expression level of transfected Z and ΔNp63α proteins, R, BMRF1, and tubulin. **B)** The original western blots used to generate the supplemental Fig 5A are shown; lanes are numbered to indicate their position in the Fig A. Lanes not used are indicated by an “I”.
(TIF)

S6 Fig P53KO NOKs-Akata were transfected with siRNAs targeting either ΔNp63α or a control sequence. After two days the cells were harvested for immunoblot analysis and the expression of ΔNp63α, Z, R, and tubulin was assessed.
(TIF)

S7 Fig The original western blots used to construct Fig 7 are shown. The lanes used in the blot shown in Fig 7 are labelled. Different western blots were used to derive the ΔNp63α, p63α, and R expression levels, and another western blot was used to derive the Z, BMRF1, and tubulin expression levels. “Blank” (B) refers to lanes where no protein was loaded. Note that the same protein lysates were used in each of the western blots shown.
(TIF)

S8 Fig **A)** The original western blots used to construct Fig 10A are shown. The lanes used in the blot shown in Fig 10A are labelled. Lanes not used in the final figure are indicated by a “I” symbol. **B)** The original western blots used to construct Fig 10C are shown on the right panel, along with Fig 10C on the left panel with lanes number 1 through 6. The lanes used in the original blots shown in Fig 10C are numbered 1 through 6. Lanes not used in the final Fig are indicated by a “I” symbol. **C) Left panel:** NOKs-Akata cells were transfected with siRNAs against c-myc or a control siRNA for two days, and then transfected with or without an R expression vector in the presence or absence of ΔNp63α. Immunoblot was performed one day later to examine expression of c-myc, R, ΔNp63α, BMRF1, and tubulin. Black lines indicate where irrelevant lanes in the blot were removed. **Right panel**: In the original western blot used to make the left panel figure, the lanes used in the figure are numbered 1 through 6, and lanes not used are indicated by a “I” symbol.
(TIF)
