# Supplementary figures and images for: ΔNp63α promotes Epstein-Barr virus latency in undifferentiated epithelial cells
Source: PLoS Pathog. 2021 Nov 8;17(11):e1010045. doi: 10.1371/journal.ppat.1010045 (PMC8601603; doi:10.1371/journal.ppat.1010045)

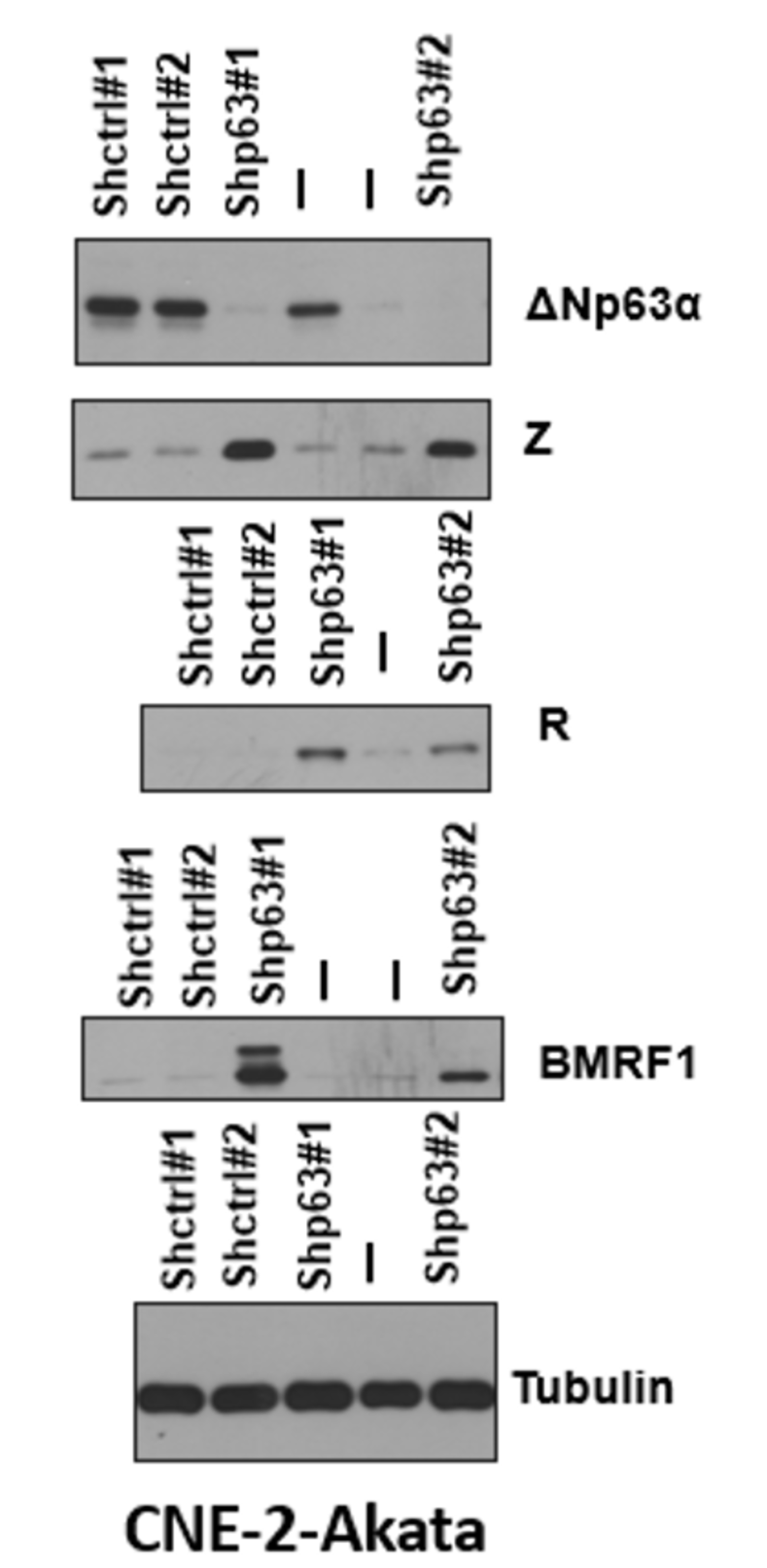

Supplement: S1 Fig — The lanes used in the blot shown in Fig 2A are labelled. Lanes not used in the final figure are indicated by a “I” symbol. (TIF) [file ppat.1010045.s001.tif]

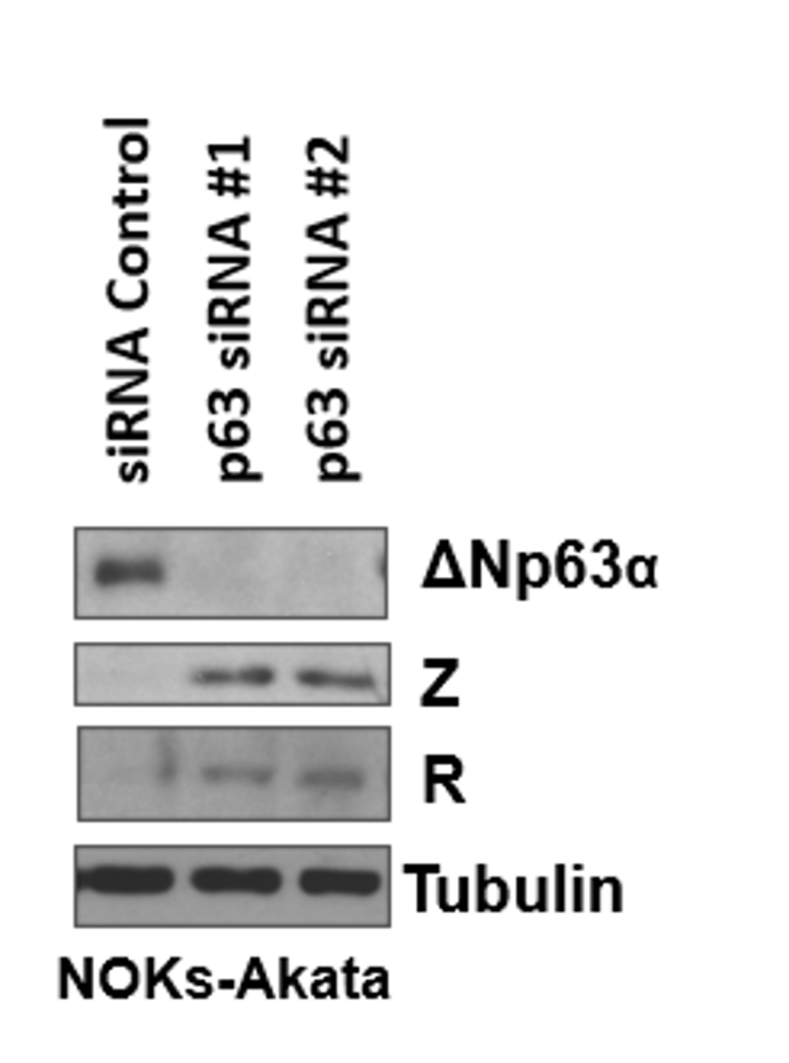

Supplement: S2 Fig — Two days after transfection the cells were harvested for immunoblot analysis and the expression of ΔNp63α, Z, R, and tubulin was determined. (TIF) [file ppat.1010045.s002.tif]

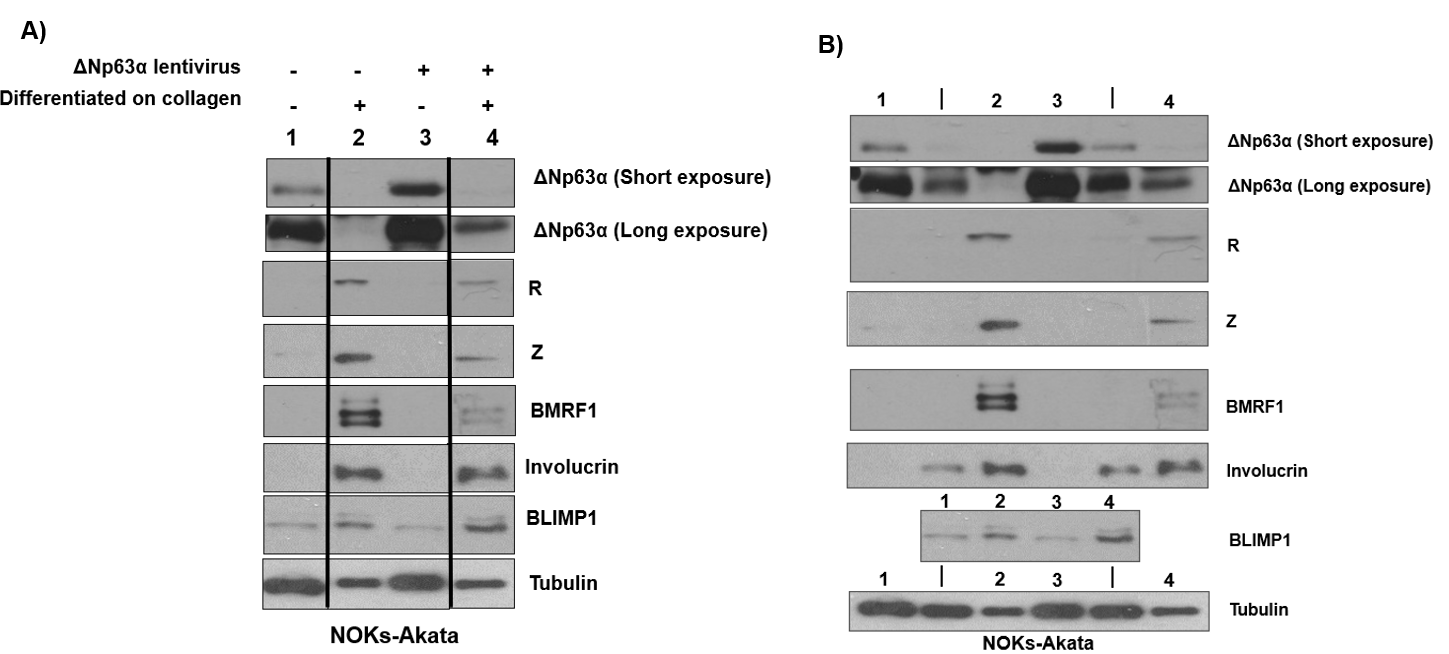

Supplement: S3 Fig — A) NOKs-Akata infected with either a lentivirus expressing ΔNp63α or a control lentivirus were differentiated on a collagen membrane or untreated for three days before harvesting for immunoblots examining the expression of ΔNp63α, R, Z, BMRF1, involucrin, BLIMP1, and tubulin. B) The original western blots used to construct supplemental Fig 3A are shown; the lanes used for the figure are labeled 1 through 4, and lanes not used are indicated by a “I” symbol. (TIF) [file ppat.1010045.s003.tif]

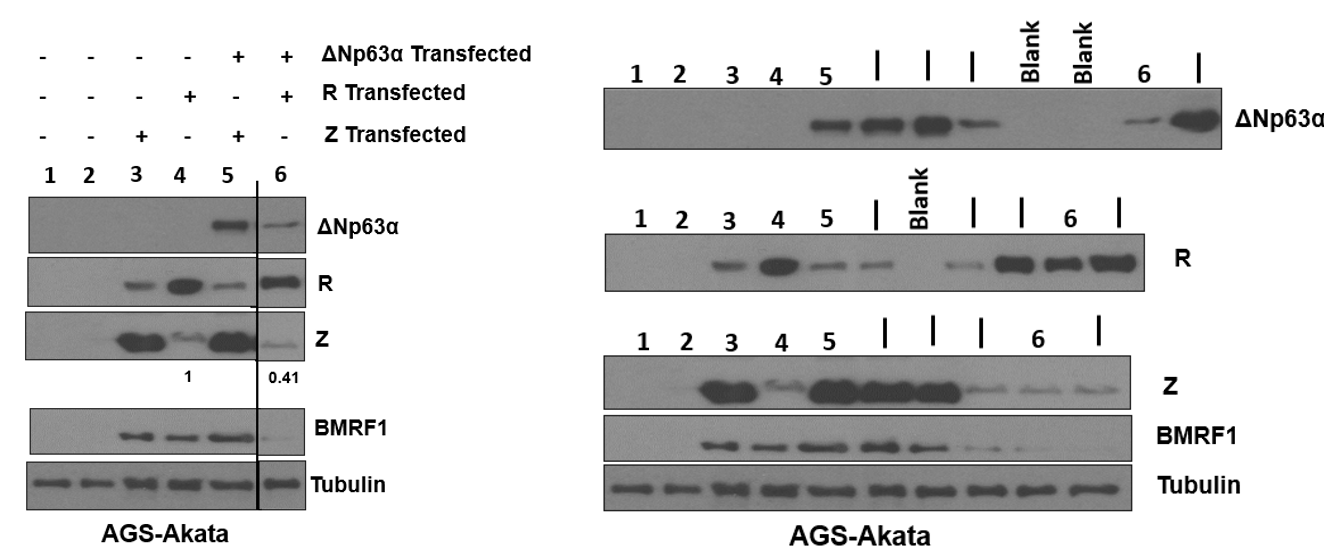

Supplement: S4 Fig — The lanes used in the blot shown in Fig 4A are labelled 1–6 as indicated. Lanes not used in the final figure are indicated by a “I” symbol. Different western blots were used to derive the ΔNp63α and R expression levels whereas the same three western blots were used to derive the Z, BMRF1, and tubulin expression levels. “Blank” refers to lanes where no protein was loaded. Note that the same protein lysates were used in each of the western blots shown. (TIF) [file ppat.1010045.s004.tif]

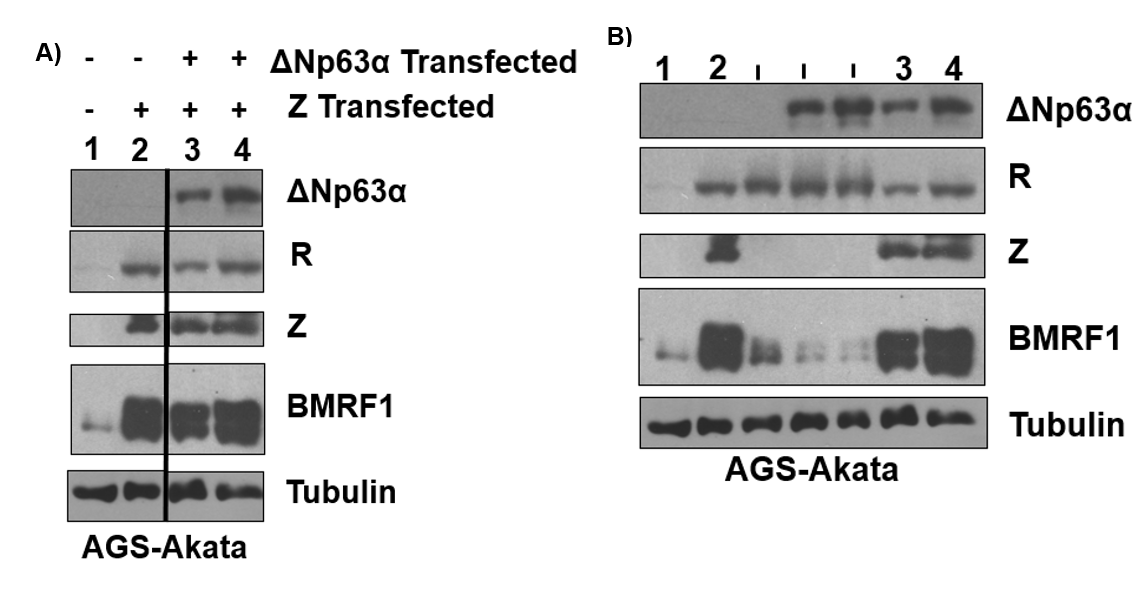

Supplement: S5 Fig — A) AGS-Akata cells were transfected with a vector control or Z expression vector in the presence of absence of ΔNp63α expression vector, as indicated. Western blots were performed to examine the expression level of transfected Z and ΔNp63α proteins, R, BMRF1, and tubulin. B) The original western blots used to generate the supplemental Fig 5A are shown; lanes are numbered to indicate their position in the Fig A. Lanes not used are indicated by an “I”. (TIF) [file ppat.1010045.s005.tif]

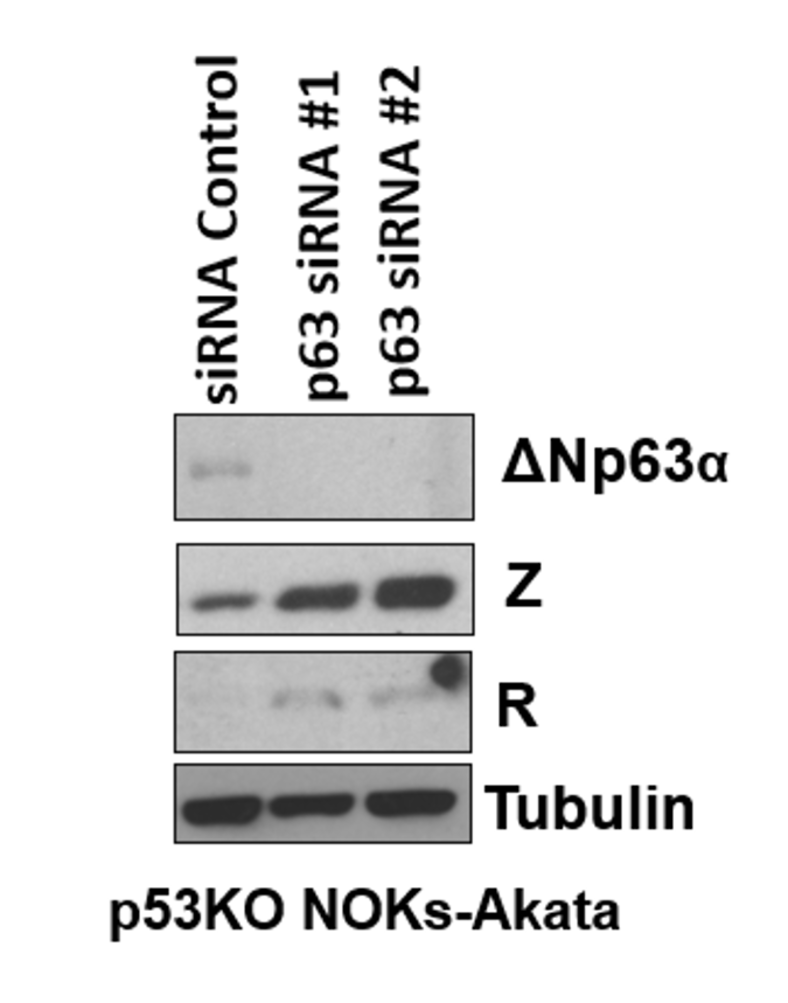

Supplement: S6 Fig — After two days the cells were harvested for immunoblot analysis and the expression of ΔNp63α, Z, R, and tubulin was assessed. (TIF) [file ppat.1010045.s006.tif]

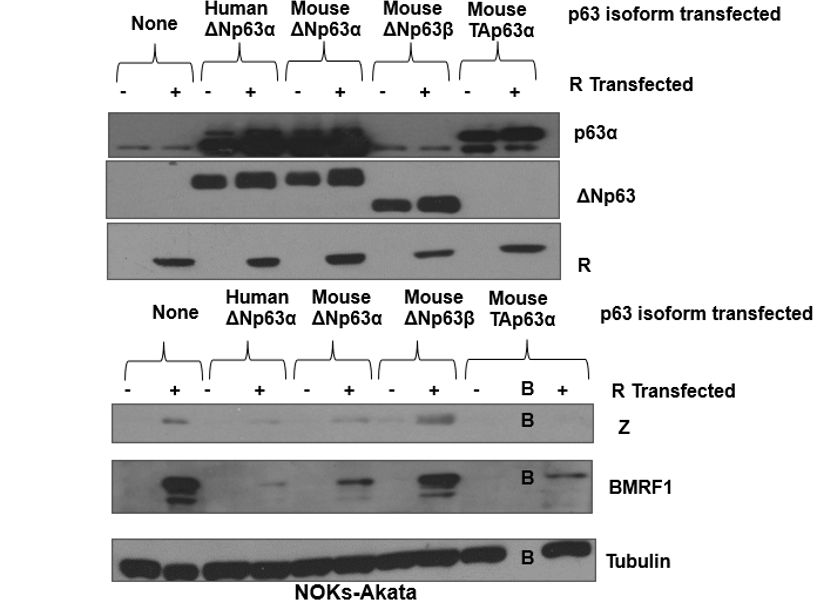

Supplement: S7 Fig — The lanes used in the blot shown in Fig 7 are labelled. Different western blots were used to derive the ΔNp63α, p63α, and R expression levels, and another western blot was used to derive the Z, BMRF1, and tubulin expression levels. “Blank” (B) refers to lanes where no protein was loaded. Note that the same protein lysates were used in each of the western blots shown. (TIF) [file ppat.1010045.s007.tif]

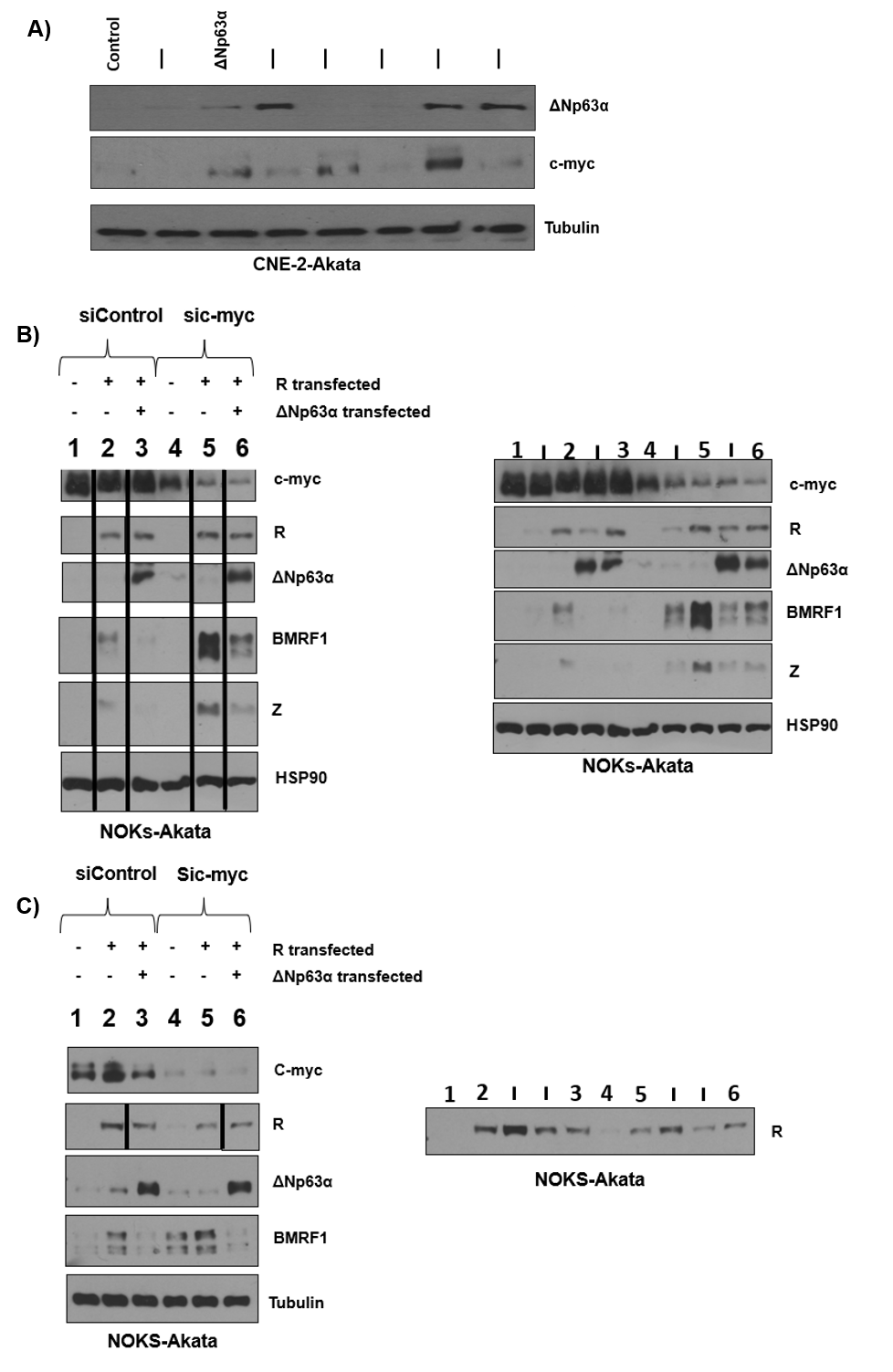

Supplement: S8 Fig — A) The original western blots used to construct Fig 10A are shown. The lanes used in the blot shown in Fig 10A are labelled. Lanes not used in the final figure are indicated by a “I” symbol. B) The original western blots used to construct Fig 10C are shown on the right panel, along with Fig 10C on the left panel with lanes number 1 through 6. The lanes used in the original blots shown in Fig 10C are numbered 1 through 6. Lanes not used in the final Fig are indicated by a “I” symbol. C) Left panel: NOKs-Akata cells were transfected with siRNAs against c-myc or a control siRNA for two days, and then transfected with or without an R expression vector in the presence or absence of ΔNp63α. Immunoblot was performed one day later to examine expression of c-myc, R, ΔNp63α, BMRF1, and tubulin. Black lines indicate where irrelevant lanes in the blot were removed. Right panel: In the original western blot used to make the left panel figure, the lanes used in the figure are numbered 1 through 6, and lanes not used are indicated by a “I” symbol. (TIF) [file ppat.1010045.s008.tif]
